# Mapping of a Pale Green Mutant Gene and Its Functional Verification by Allelic Mutations in Chinese Cabbage (*Brassica rapa* L. ssp. *pekinensis*)

**DOI:** 10.3389/fpls.2021.699308

**Published:** 2021-08-12

**Authors:** Yonghui Zhao, Shengnan Huang, Meidi Zhang, Yun Zhang, Hui Feng

**Affiliations:** Department of Horticulture, Shenyang Agricultural University, Shenyang, China

**Keywords:** Chinese cabbage, pale green, allelic mutations, cloning, DVR

## Abstract

Leaves are the main organ for photosynthesis, and variations in leaf color affect photosynthesis and plant biomass formation. We created two similar whole-plant pale green mutants (*pem1* and *pem2*) from the double haploid (DH) Chinese cabbage line “FT” through ethyl methanesulfonate (EMS) mutagenesis of seeds. Photosynthetic pigment contents and net photosynthetic rates were significantly lower in the mutants than in the wild-type “FT,” and the chloroplast thylakoid endomembrane system was poor. Genetic analysis showed that the mutated phenotypes of *pem1* and *pem2* were caused by a single nuclear gene. Allelism tests showed that *pem1* and *pem2* were alleles. We mapped *Brpem1* to a 64.25 kb region on chromosome A10, using BSR-Seq and map-based cloning of 979 F_2_ recessive individuals. Whole-genome re-sequencing revealed a single nucleotide polymorphism (SNP) transition from guanine to adenosine on *BraA10g021490.3C* in *pem1*, causing an amino acid shift from glycine to glutamic acid (G to E); in addition, *BraA10g021490.3C* in *pem2* was found to have a single nucleotide substitution from guanine to adenosine, causing an amino acid change from E to lysine (K). *BraA10g021490.3C* is a homolog of the *Arabidopsisdivinyl chlorophyllide a 8-vinyl-reductase* (*DVR*) gene that encodes 3,8-divinyl protochlorophyllide a 8-vinyl reductase, which is a key enzyme in chlorophyll biosynthesis. Enzyme activity assay and chlorophyll composition analysis demonstrated that impaired DVR had partial loss of function. These results provide a basis to understand chlorophyll metabolism and explore the mechanism of a pale green phenotype in Chinese cabbage.

## Introduction

Leaf color mutations that are widespread in the plant kingdom have been identified in many plants, such as maize (Lonosky et al., 2004), rice (Oster et al., 2000; Jung et al., 2003; Lee et al., 2005), barley (Preiss and Thornber, 1995; Rudoi and Shcherbakov, 1998), and *Arabidopsis* (Carol et al., 1999; Kumar et al., 2000). The typical variation in plant leaf color is yellowing, which has become an important material for studying the mechanism of photosynthesis, chlorophyll biosynthetic pathways, chloroplast development, and its genetic control mechanisms (Hansson et al., 1999; Zhao et al., 2008). The color of plant leaves is affected by genetic and environmental factors, and is determined by various pigments, including chlorophyll, carotenoids, and anthocyanins (Li et al., 2019). Chlorophyll, the main pigment in photosynthesis, can absorb most of the red and purple light, and thus plays a key role in light absorption during photosynthesis and affects plant yield to some extent (Fromme et al., 2003). However, chlorophyll biosynthesis is a complex process involving many compounds. The chlorophyll biosynthetic pathway in higher plants consists of 16 enzymatic reactions involving 15 enzymes (Beale, 1999; Wang et al., 2010b). Chlorophyll is produced from L-glutamate in a sequential manner through the formation of protoporphyrin IX, Mg-protoporphyrin IX, protochlorophyllide a, and, finally, chlorophyll a and chlorophyll b via the tetrapyrrole biosynthetic pathway (Tanaka et al., 1998; Beale, 1999; Lange and Ghassemian, 2003). In *Arabidopsis thaliana*, the chlorophyll biosynthetic pathway is encoded by 27 genes; all of which have been cloned (Beale, 2005; Nagata et al., 2005). Many yellow mutants are generated by mutations in the genes involved in the chlorophyll biosynthesis pathway, which encode key enzymes, such as glutamyl tRNA reductase (GluTR), Mg-chelatase, and chlorophyll a oxidase (CAO) (Oster et al., 2000; Kim et al., 2005; Lee et al., 2005; Gao et al., 2016; Fu et al., 2019).

Classified on the number of vinyl side chains, there are two types of chlorophyll: monovinyl chlorophyll (MV-Chl) and divinyl chlorophyll (DV-Chl) (Nagata et al., 2005; Wang et al., 2010b), which are both accumulated in various organisms (Kim and Rebeiz, 1996; Rebeiz et al., 2003; Islam et al., 2008). To date, five DV-Chl derivatives have been identified, namely, DV-Mg-Proto (Kim and Rebeiz, 1996), DV-Mg-Proto IX ester (MPE) (Kolossov et al., 2006), DV-Pchlide a (Tripathy and Rebeiz, 1988), DV-Chlide a (Parham and Rebeiz, 1992, 1995), and DV-Chl a (Adra and Rebeiz, 1998). The DV-Chl derivative is formed first, and then the MV-Chl derivative is converted by 3,8-divinyl protochlorophyllide a 8-vinyl reductase (DVR) to the corresponding DV-Chl derivative (Nagata et al., 2005; Wang et al., 2010b). Almost all photosynthetic aerobic organisms in the biosphere use MV-chlorophyll to absorb light energy for photosynthesis (Chisholm et al., 1992; Porra, 1997). Only a few species (*Prochlorococcus marinus*) with a distinct photosynthetic antenna system can use DV-chlorophyll for photosynthesis (Partensky et al., 1999; Ito et al., 2008). Phylogenetic and genomic findings suggest that the progenitors of *Prochlorococcus* lost the *DVR* gene and acquired the ability to utilize DV-chlorophyll during evolution (Nagata et al., 2005). The *DVR* gene has been identified in only a few species to date, including *A. thaliana*, rice, maize, cucumber, and green sulfur bacteria. Nakanishi et al. (2005) and Nagata et al. (2005) independently identified *DVR* in chlorophyll content-reduced mutants as *AT5g18660*, which is the last identified gene required for chlorophyll biosynthesis in higher plants. However, a *DVR* gene in Brassica plants has not been identified.

Chinese cabbage (*Brassica campestris* L. ssp. *pekenensis*) is the main winter vegetable in northern Asia. Leaf color is related to photosynthesis and product quality of Chinese cabbage, so breeders have always focused on its leaf color variations. However, there are few reports about the cloning and identification of the leaf color gene in Chinese cabbage. We identified two similar pale green mutants (*pem1* and *pem2*) from a Chinese cabbage mutant library through ethyl methanesulfonate (EMS) mutagenesis of germinating seeds. Genetic and photosynthetic characteristics of the mutants *pem1* and *pem2* had been analyzed. *BraA10g021490.3C*, a homolog of Arabidopsis *DVR*, encoding 3,8 divinyl protochlorophyllide a 8-vinyl reductase, was considered as a responsible gene of *Brpem1* and *Brpem2*. The mutants could be used as material to investigate chlorophyll metabolism and explore the mechanism that leads to the pale green phenotype in Chinese cabbage.

## Materials and Methods

### Plant Materials

The mutant *pem1* and *pem2* lines were obtained from the “FT” line, which is a double haploid (DH) Chinese cabbage generated via microspore cultures after treating the seeds of the “FT” Chinese cabbage line with EMS. The mutants were crossed with wild-type “FT” to construct F_1_, F_2_, and BC_1_ populations for genetic analysis, and with K23, a Pak choi inbred line, which has great difference with the mutant in characteristics, to construct F_2_ segregation populations for gene mapping. All plants were grown in a greenhouse or open ground at Shenyang Agriculture University.

### Determination of Photosynthetic Pigments

Chlorophyll was extracted from fresh fifth true leaves (0.1 g), using 80% (V/V) ethanol in acetone in darkness for 24 h. Thereafter, the absorbance of extract was measured at 470, 645, and 663 nm, using a DU800 Ultraviolet Spectrophotometer (Beckman Coulter, La Brea, CA, USA). The control was 80% (V/V) ethanol in acetone. The total chlorophyll, chlorophyll a, chlorophyll b, and carotenoid contents in the plants were calculated as described by Holm (1954). Three biological repeats were determined for each material, and three times the technical repeats were performed in each biological repeat.

### Determination of Photosynthetic Characteristics

The photosynthetic characteristics of the fifth true leaves of mutant and wild-type (WT) plants at the same growth stage were assessed during a clear morning. Net photosynthetic rates (PN; μmol CO_2_ m^−2^s^−1^), stomatal conductance (GS; mol H_2_O m^−2^s^−1^), intercellular carbon dioxide concentrations (CI; μmol CO_2_ mol^−1^), and transpiration rates (TS; mmol H_2_O m^−2^s^−1^) were measured, using an Li-6400 (Li COR Biosciences, Lincoln, NE, USA). Three biological repeats were determined for each material, and three times the technical repeats were performed in each biological repeat.

### Observation of Chloroplast Ultrastructure

Fifth true leaves of 6-week-old plants (~5 × 1 mm) were fixed in 3% glutaraldehyde at 4°C overnight, washed four times with a 1% phosphoric acid buffer, and then placed in osmium tetroxide (1%) for 2 h. After three washes with the 1% phosphoric acid buffer, the tissues were dehydrated, using graded series of 50, 70, and 80% ethanol, followed by 90 and 100% acetone for 24 h, and then epoxy resin Epon812 was used for encapsulation and polymerization. The tissues were sectioned, using a Leica EM UC7 Ultra-Thin Microtome (Leica Microsystems GmbH, Wetzlar, Germany) and stained with uranyl acetate and lead citrate. Chloroplast ultrastructure was examined, using an HT7700 transmission electron microscope (Hitachi Ltd., Tokyo, Japan) at Analysis and Test Center of Shenyang Agricultural University.

### BSR-Seq (Bulked Segregant RNA-Sequencing)

The F_2_ population derived from Chinese cabbage pale green mutant *pem1* crossed with Pak choi K23 was used to construct mutant-phenotype pool (MPpool) and wild-type phenotype pool (WPpool) for BSR-Seq. Total RNA was, respectively, extracted from the third true leaves of pale green and green plants, using the plant total RNA extraction kit (Tiangen Biotech, Beijing, China).

The two mixed RNA pools were sequenced, using an Illumina HiSeq (Illumina Inc., San Diego, CA, USA). The quality of the original transcriptome sequencing data was evaluated, using fastQC (version 0.10.1) and preprocessed, using Cutadapt (version 1.9.1) to screen out low-quality data and remove contaminants and joint sequences. Subsequently, screened data were mapped on the reference genome (http://brassicadb.org/brad/datasets/pub/Genomes/Brassica_rapa/V3.0/) using HISAT (v2.0.14). Transcripts predicted by StringTie (v1.0.4) were classified by ASprofile (v1.0.4), and their expression was analyzed. Finally, transcriptional assembly and single nucleotide variants (SNV) were analyzed. Mutant loci with sequencing coverage greater than 3X in the MP-pool and WP-pool were screened, and the Euclidean distance^5^ (ED^5^) values of SNV were calculated to eliminate background noise. According to the ED^5^ value to rank, the top 1% was taken as the threshold to identify chromosome regions linked to the target traits (Tan et al., 2019). The raw sequence data of the BSR-Seq are available from the Sequence Read Archive at NCBI under the accession No. SRR14339734.

### Whole-Genome Re-Sequencing

The total genomic DNA of wild-type “FT” and mutant *pem1* was extracted, using a DNA Secure Plant Kits (Tiangen, Beijing, China). A DNA library was constructed with an insert fragment of 400 bp, and the paired-end (PE) of the library was sequenced, using next-generation sequencing (NGS) based on a NovaSeq 6000 System sequencer (Illumina, San Diego, USA). The coverage of whole-genome re-sequencing was 25x, and the sequencing mode was paired-end, 2 × 150 bp. High-quality data were generated from the raw data by removing 3' end joint contamination, using Adapter Removal (version 2); quality filtering, using the sliding window method; and length filtering. By mapping to the reference genome, we detected SNP (single nucleotide polymorphism) and INEDL (insertion and deletion), CNV (copy number variation), SV (structural variation of chromosome), using GATK (Zhu et al., 2015), CNVnator v0.2.7 (Abyzov et al., 2011), Breakdancer 1.3.7 (Fan et al., 2014), respectively. The SNP/INDEL loci were annotated, using ANNOVAR (Wang et al., 2010a). The raw sequence data of the whole-genome re-sequencing are available from the Sequence Read Archive at NCBI under the accession numbers SRR14340669 and SRR14340670.

### DNA Extraction, PCR, and Marker Development

Total genomic DNA extracted from plant leaves, using the modified CTAB method, was amplified, using Mastercycler^®^ nexus GSX1 (Eppendorf AG., Hamburg, Germany). The primers used for PCR amplification were shown in Supplementary Table S2. The reaction volume was 10 μL, and the conditions were 95°C, 5 min, followed by 35 cycles of 95°C for 30 s; 56°C for 30 s, then final extension at 72°C for 5 min.

The sequences of BSR-Seq target regions were downloaded from the Brassica database. The SSR and INDEL primers were designed, using Primer Premier 5.0 software (Premier Inc., Charlotte, USA) and synthesized by GENEWIZ (Tianjin, China). Polymorphisms in the primers were screened between the parent mutants *pem1* and K23, using polyacrylamide gel electrophoresis.

### Linkage Analysis and Genetic Map Construction

The recombinants from recessive individuals in the F_2_ segregation population were screened, using polymorphic primers between parents to analyze linkage. According to the recombination frequency between polymorphic markers and mutant genes, a genetic linkage map between molecular markers and mutant genes was constructed, using Mapmaker V. 3.0 (Whitehead Institute, Cambridge, MA, USA).

### Clone Sequencing

The full-length and promoter sequences of candidate genes were obtained by cloning and sequencing. Primers were designed according to the gene sequence information and are shown in Supplementary Table S5. PCR products were purified, using a Gel Extraction Kit (Omega Bio-Tek Inc., Norcross, GA, USA), then ligated to pGEM^®^-T Easy Vector (Promega, USA) and transformed into Top 10 competent cells (CWBIO, Beijing, China). The positive clones were screened as blue and white colonies in the Xgal-IPTG medium and sequenced by GENEWIZ (Tianjin, China), using 3730 × lDNA Analyzer (Applied Biosystems, Foster City, CA, USA). Sequences were aligned, using DNASTAR (DNASTAR, Inc., Madison, WI, USA).

### Bioinformatic Analysis of BrDVR

The sequence and the information of *BrDVR* were downloaded from the Brassica database. The conserved domain of BrDVR was analyzed online at NCBI (https://www.ncbi.nlm.nih.gov/Structure/cdd/wrpsb.cgi). Protein localization was predicted, using the TargetP-2.0 (http://www.cbs.dtu.dk/services/TargetP/) server and chloroplast transit peptides (cTP) in protein sequences, and the locations of potential cTP cleavage sites were predicted, using ChloroP (http://www.cbs.dtu.dk/services/ChloroP/). Three-dimensional structures of the proteins were built, using SWISS-MODEL (https://swissmodel.expasy.org/interactive/). The amino acid sequence and accession numbers of BrDVR homologs were obtained from NCBI BLAST. Sequence alignment was performed, using DNAMAN V6 (Lynnon BioSoft, Canada). A phylogenetic tree was constructed with MEGA-X (Kumar et al., 2018), using Clustal W and neighbor joining based on 1,000 bootstrap replications.

### Verification of Gene Expression Using qRT-PCR

The expression of candidate genes was detected, using quantitative real-time (qRT)-PCR. Total RNA was extracted from the leaves of mutant and wild-type plants, using the plant total RNA extraction kit (Tiangen, Beijing, China). Single-stranded RNA was reverse transcribed into cDNA, using FastQuant RT Super Mix (Tiangen, Beijing, China); gene-specific primers designed using Primer Premier 5.0 (Supplementary Table S6) were used for qRT-PCR with SYBR Green PCR Master Mix (Takara Bio Inc., Kusatsu, Japan). The reaction mix (20 μL) included 10 μL of 2 × UltraSYBR mixture, 0.8 μL of diluted cDNA, 0.4 μL of forward and reverse primers, and 8.4 μL of double-distilled H_2_O. The *ACTIN* gene was used as internal standard, and the reaction proceeded under the following conditions: 95°C for 10 min, followed by 40 cycles at 95°C for 15 s, 60°C for 1 min, 95°C for 15 s, 60°C for 1 min, 95°C for 15 s, and 60°C for 15 s. All reactions included three biological and two technical replicates.

### Enzyme Activity Assays

DVR activity was evaluated, using the Plant DVR^®^ ELISA Kit (Meimian Biotech Co., Ltd., Jiangsu, China) via a double antibody sandwich method. Fresh leaf samples of 6-week-old (0.1 g) were homogenized in 1 ml of PBS (pH 7.4) and centrifuged at 12,000 × *g* for 20 min; the supernatant was then collected. The kit assay DVR activity in the sample used Purified Plant DVR antibody to coat microtiter plate wells, made solid-phase antibody, then added a DVR sample to the wells, combined antibody, which, with HRP labeled, became antibody-antigen-enzyme-antibody complex, and, after washing completely, added TMB substrate solution. TMB substrate became blue color at HRP enzyme-catalyzed, reaction was terminated by the addition of a sulfuric acid solution, and the color change was measured, using spectrophotometer at a wavelength of 450 nm. The enzyme activity of DVR in the samples was then determined by comparing the O.D. of the samples to the standard curve. Three biological repeats were determined for each material, and three times the technical repeats were performed in each biological repeat.

### HPLC Analysis for Chlorophyll Composition

Chlorophyll used for HPLC analysis was extracted from fresh leaf samples of 6-week-old *pem1, pem2*, and “FT” plants, using 100% acetone. After centrifuged at 12,000 × *g* for 15 min, the supernatant was dried under N_2_ gas, redissolved in acetone, and filtered with 0.22 μm organic filter membrane. HPLC analysis was performed, using an Alliance e2695 instrument with a C8 column (4.6 mm i.d. × 250 mm long; 5 μm; Agilent) according to the method of Zapata et al. (2000), using an isocratic system, consisting of methanol: acetonitrile: acetone at 1:3:1 at 40°C and a flow rate of 1 m min^−1^. Eluted samples were monitored, using a 2,489 UV/Vis detector at 660 nm. Chl *a* and *b* standards (Sigma, St. Louis, USA) were used as controls. Three biological repeats were determined for each material, and three times the technical repeats were performed in each biological repeat.

### Statistical Analysis

The significance difference between the mutants and wild type “FT” was analyzed by Analysis of Variance (ANOVA) of software SPSS (IBM, Armonk, USA) at the significance level of 0.05.

## Results

### Mutant Creation and Phenotypic Characterization

In order to further study the functional genome of Chinese cabbage, DH line “FT,” which was generated by microspore culture as the WT to create a Chinese cabbage mutant library. Treated 7,800 “FT” germinated seeds in 0.8% EMS for 12 h, to obtain 701 mutant M2 lines, including 286 yellow mutants. Among which, seven mutants with similar phenotype were investigated, and their allelism test (see as the section of “The Allele Test of *Brpem1* and *Brpem2*”) revealed that the mutant genes of *pem1* and *pem2* were allele, and *pem1* and *pem2* were chosen for further investigation.

**Figure 1:**
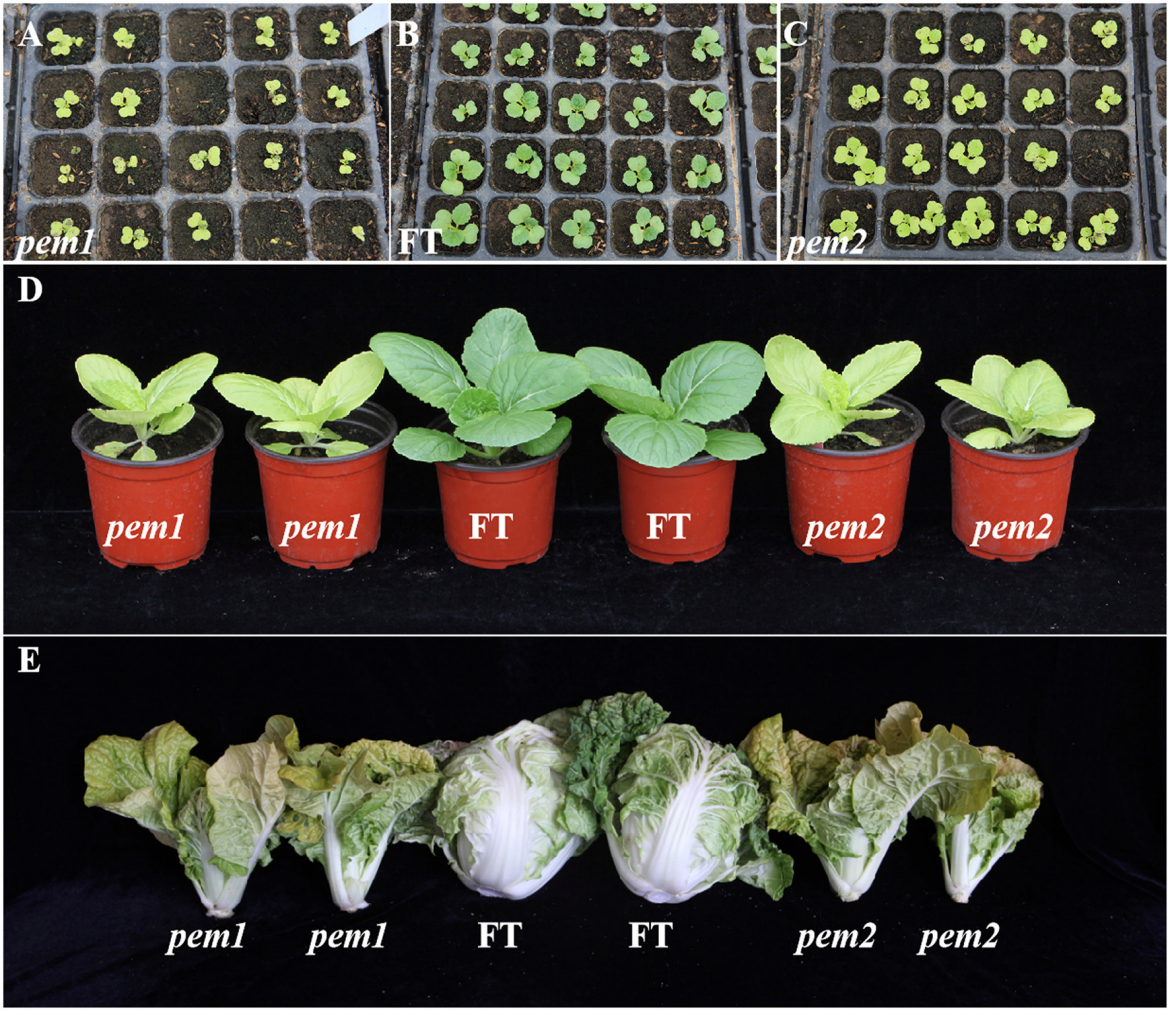
Phenotypic characterization of *pem1, pem2*, and “FT.” **(A-C)** Phenotypic characterization of *pem1* **(B)** and *pem2* **(C)** compared with “FT” **(A)** at cotyledons stage. **(D)** Phenotypic characterization of *pem1* and *pem2* compared with “FT” at the seedling stage. **(E)** Phenotypic characterization of *pem1* and *pem2* compared with “FT” at the heading stage.

The mutant lines *pem1* and *pem2* exhibited the plant pale green phenotype since the cotyledon stage persisted until the heading stage (the leafy head formation stage) (Figure 1). The pale green phenotype was evident in all the aboveground organs. The mutant plants were weak and grew so slowly that they could not head during the heading stage; however, pollination and seed setting were normal.

### Analysis of Photosynthetic Pigments

The contents of chlorophyll a, chlorophyll b, total chlorophyll, and carotenoids were significantly decreased in *pem1* and *pem2* compared with “FT,” and the total Chl contents were, respectively, decreased by 57.49 and 54.54% (Table 1). The ratios of chlorophyll a content to chlorophyll b content (Chla/b) and of carotenoids content to total chlorophyll content (Car/Chl) were significantly increased (Table 1). Therefore, the chlorophyll content showed a higher decrease than the carotenoid content, and the decrease was greater for chlorophyll b content than chlorophyll a content. Previous studies have shown that chlorophyll is responsible for the green pigmentation in plants, and the presence of carotenoids affects leaf color (Wu et al., 2018; Sun et al., 2020). We speculated that this significant change in the photosynthetic pigment was the direct cause of the pale green phenotype in *pem1* and *pem2*.

**Table 1:** Content of photosynthetic pigments in *pem1* and *pem2* compared with wild-type “FT” (mgg-1).

**Material**	**Chla (ratio of mutant to “FT”)**	**Chlb (ratio of mutant to “FT”)**	**Total Chl (ratio of mutant to “FT”)**	**Chla/b**	**Car (ratio of mutant to “FT”)**	**Car/Chl**
“FT”	13.51 ± 0.34^a^	4.79 ± 0.41^a^	18.30 ± 0.60^a^	2.86 ± 0.25^b^	4.51 ± 0.49^a^	0.25 ± 0.03^b^
*pem1*	6.30 ± 0.59^b^ (46.63%)	1.48 ± 0.03^b^ (30.90%)	7.78 ± 0.62^b^ (42.51%)	4.25 ± 0.31^a^	2.46 ± 0.23^b^ (54.55%)	0.32 ± 0.01^a^
*pem2*	6.71 ± 0.22^b^ (49.67%)	1.61 ± 0.11^b^ (33.61%)	8.32 ± 0.11^b^ (45.46%)	4.22 ± 0.44^a^	2.54 ± 0.21^b^ (56.32%)	0.31 ± 0.02^a^

*Values are shown as the means ± standard error (SE) from three independent determinations. Superscript letters followed by figures represent significant differences at 5% (ANOVA)*.

### Defective Chloroplast Structure and Inefficient Photosynthesis

**Figure 2:**
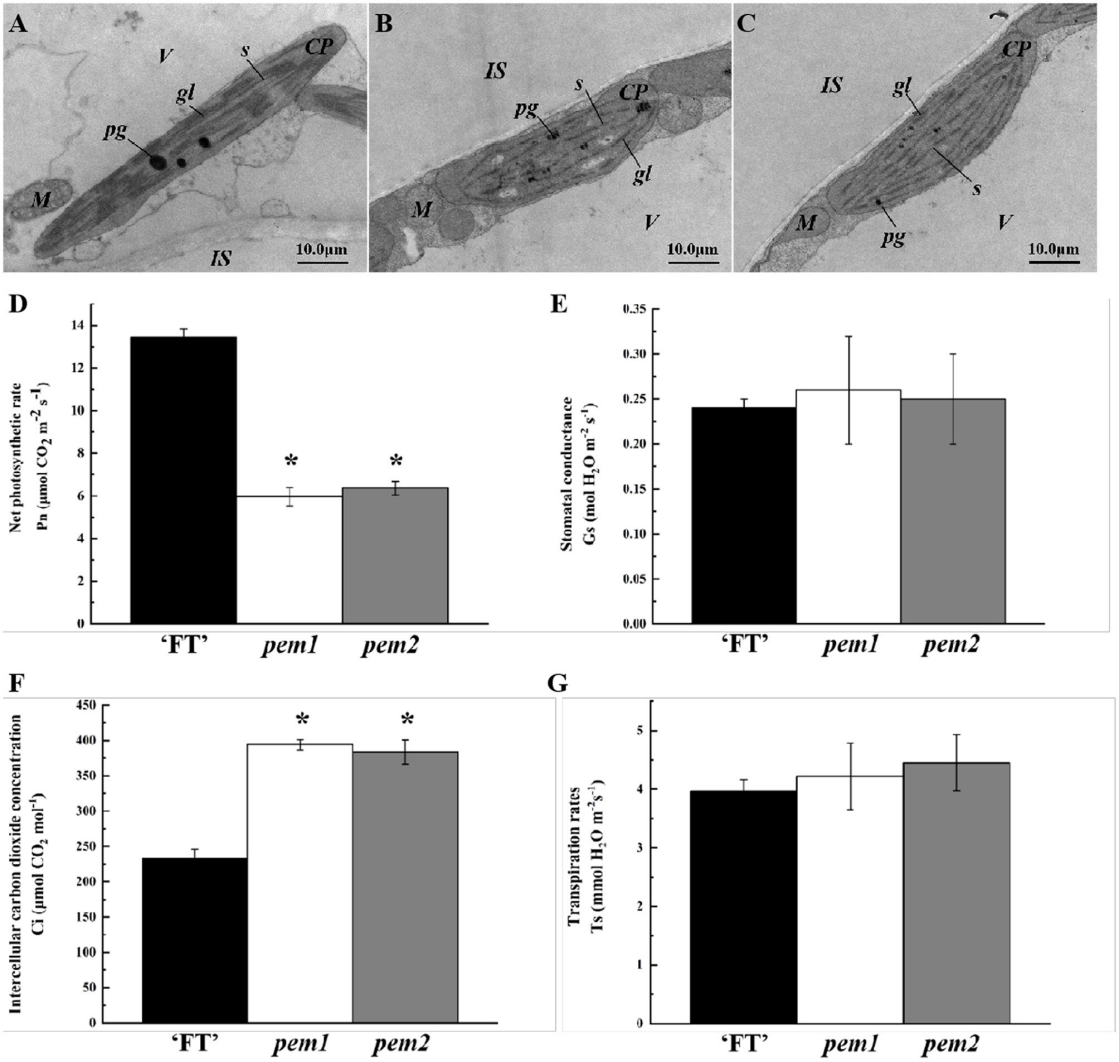
Ultrastructure of chloroplasts and photosynthetic characteristics in *pem1* and *pem2* compared with “FT” in Chinese cabbage. Mesophyll cells of “FT” **(A)**, *pem1* **(B)**, and *pem2* **(C)** were visualized with TEM (magnification, × 50,000). *cp*, chloroplast; *gl*, grana lamella; *is*, intercellular space; *m*, mitochondria; *pg*, plastoglobule; *s*, stroma; *v*, vacuole. Scale is shown at the bottom right. Photosynthetic characteristics: net photosynthetic rates **(D)**, stomatal conductance **(E)**, intercellular carbon dioxide concentrations **(F)**, and transpiration rates **(G)** of *pem1* and *pem2* compared with the wild-type “FT.” ^*^indicate significant differences at 5% (ANOVA).

Chloroplast development in *pem1* and *pem2* was investigated, using transmission electron microscopy (TEM). The chloroplasts in “FT” had a clear internal structure, with a well-developed somatic membrane system, and many closely arranged stacks of grana with rich lamellae (Figure 2A), whereas those in *pem1* and *pem2* were slightly smaller and had a poor internal structure, singular grana lamellae, and no distinct grana stacks (Figures 2B,C). Therefore, we speculated that the mutation affected the chloroplast development.

Photosynthetic parameters significantly differed between “FT” and the two mutant lines. Compared with “FT,” the net photosynthetic rate of *pem1* and *pem2* decreased significantly by 55.65 and 52.67%, respectively (Figure 2D). The stomatal conductance and transpiration rates of the mutants did not significantly change, while the intercellular CO_2_ concentrations of *pem1* and *pem2* significantly increased by 69.34 and 65.04%, respectively (Figures 2E–G). This indicated that the mutant had a weak ability to fix CO_2_, which might be related to the lower chlorophyll content.

### Analysis of Genetic Characteristics

We constructed seven generation populations to clarify the genetic characteristics of mutant *pem1*. Table 2 shows stable pale green and the green phenotype in *pem1* and “FT,” respectively. The *pem1* mutant and “FT” reciprocal cross F_1_ plants were green, indicating that the pale green mutant features were due to an inherited nuclear recessive gene. The F_2_ population showed character separation, and the segregation ratio was 3:1 for green:pale green plants (χ^2^ < χ^0.052^ = 3.84), which corresponded to the Mendelian laws. Moreover, the BC_1_ (F_1_ × “FT”) population was green, whereas the BC_1_ (F_1_ × *pem1*) population had the character separation of 1:1 for green:pale green plants (χ^2^ < χ^0.052^ = 3.84). These results confirmed that the mutation phenotype of *pem1* was controlled by a single nuclear recessive gene. Table 2 also shows the population phenotype of *pem2*. The results indicate that the pale green phenotype of *pem2* was caused by the recessive mutation of a single nuclear gene.

**Table 2:** Genetic analysis of mutant *pem1* and *pem2* in Chinese cabbage.

**Generation**	**Green-colored plant**		**Yellow-colored plant**		**Total**		**Segregation ratio**		**χ** ^**2**^ **-test**	
	***pem1***	***pem2***	***pem1***	***pem2***	***pem1***	***pem2***	***pem1***	***pem2***	***pem1***	***pem2***
P_1_(“FT”)	78	94	0	0	78	94				
P_2_(*pem1* or *pem2*)	0	0	178	232	178	232				
F_1_(P_1_×P_2_)	23	14	0	0	23	14				
F_1_(P_2_×P_1_)	11	17	0	0	11	17				
BC_1_(F_1_×P_1_)	45	22	0	0	45	22				
BC_1_(F_1_×P_2_)	10	23	9	20	19	43	1.11:1	1.15:1	0.053 (1:1)	0.209 (1:1)
F_2_	25	88	78	25	103	113	3.12:1	3.53:1	0.023 (3:1)	0.425 (3:1)

### The Allele Test of *Brpem1* and *Brpem2*

**Figure 3:**
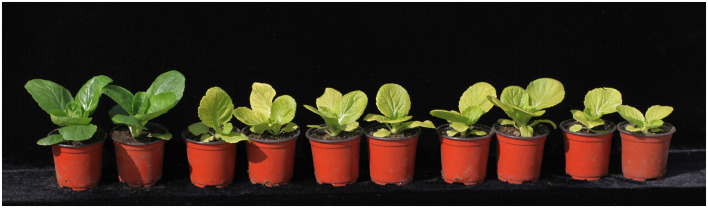
Morphological appearance of allele mutant lines (*pem1* and *pem2*). **Left** to **right**: 1 and 2, “FT”; 3 and 4, *pem1*; 5 and 6, *pem1* × *pem 2*; 7 and 8, *pem2* × *pem1*; 9 and 10, *pem2*.

Among 286 yellow mutants in the mutant library, we found seven mutants with a similar phenotype and inheritance. In order to test its allelism, we carried out semi-diallel crossing experiments. If the phenotype of the hybrid between two parents remains the mutant phenotype, the two parents are allele mutation. It was found that the hybrid between *pem1* and *pem2* showed pale green (Figure 3), which confirmed that the mutant genes of *pem1* and *pem2* were allele.

### *Brpem1* Location on Chromosome A10 Determined by BSR-Seq

**Figure 4:**
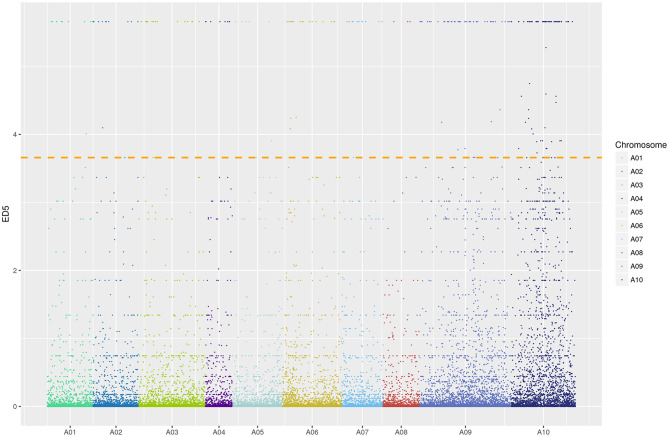
Distribution of ED^5^ values on chromosomes. Colors indicate various SNV sites on different chromosomes. Color width on X-axis represents the number of SNV sites in each chromosome; ordinate, ED^5^ of each SNV locus; a dotted line, a correlation threshold of top 1% ED^5^ value.

We constructed two mixed pools with extreme phenotypes (MP-pool and WP-pool) to identify possible regions of *pem1*, using BSR-Seq. We mapped 110,869 SNV to the Chiifu reference genome that differed between MP-pool and WP-pool. A distribution map of the ED^5^ value of different SNV on chromosomes revealed a peak in chromosome A10 (Figure 4). Two intervals were obtained on chromosome A10 (A10: 7.55–9.56, 10.71–15.35 Mb; Supplementary Table S1) according to the top 1% ED^5^ value threshold.

Based on the reference genome and the BSR-Seq candidate region, 40 pairs of SSR markers were developed. Among these, 13 markers were polymorphic between mutant line *pem1* and Pak choi K23 and produced stable, clear amplification bands (Supplementary Figure S1). Thirty recessive plants were identified in the F_2_ population to verify whether chromosome A10 is linked to the pale green trait. We found that SSRA1-1 and SSRA2-16 were linked to and located on both sides of *Brpem1* (Supplementary Table S2 and Supplementary Figure S2). Therefore, *Brpem1* was mapped to an approximately 1.7-Mb region on chromosome A10 between SSRA1-1 and SSA2-16 at estimated genetic distances of 8.27 and 1.48 cm, respectively.

### Fine Mapping of *Brpem1* by Linkage Analysis

**Figure 5:**
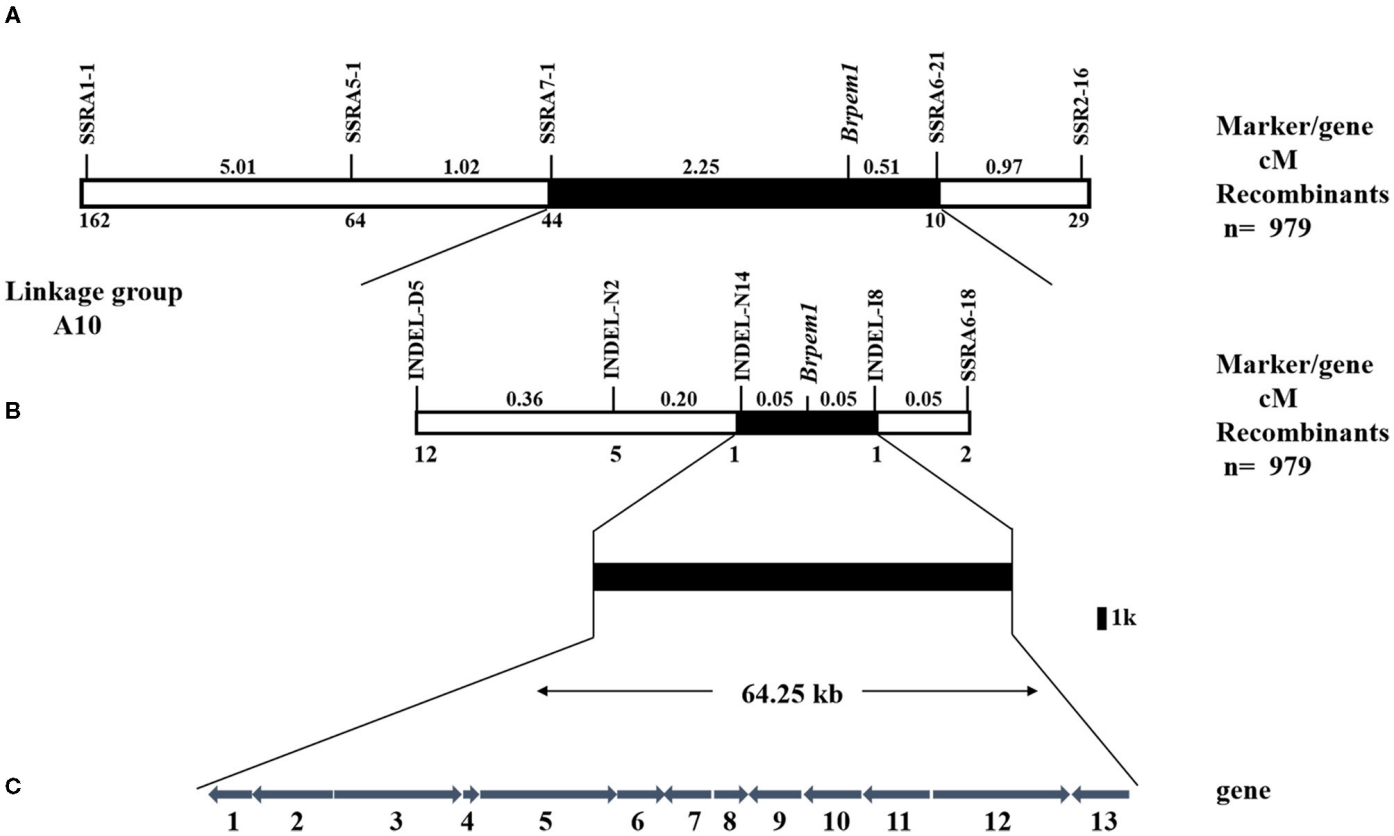
Genetic and physical maps of *Brpem1* gene locus and candidate gene analysis of *B. rapa*. A genetic linkage map of chromosome A10 was constructed, using 979 F_2_ recessive individuals with a pale green phenotype. **(A)** *Brpem1* initially located between molecular markers SSRA1-1 and SSRA2-16 in the A10 linkage group. **(B)** *Brpem1* fine mapped to the 64.25 kb region on chromosome A10 between INDEL-N14 and INDEL-I8. Numbers of recombined individuals between *Brpem1* and the marker are shown at the bottom of the linkage map. Number (cM) at the top of the linkage map, genetic distance between two adjacent markers. **(C)** The candidate region of *Brpem1* and 13 predicted genes (http://brassicadb.org/brad/datasets/pub/Genomes/Brassica_rapa/V3.0/). Numbers 1–13, order of genes in the candidate region; arrows, direction of gene expression. Supplementary Table S3 shows details of these genes.

The mapping populations were expanded to 979 individuals, and more SSR and INDEL markers between SSRA1-1 and SSA2-16 were developed to map *Brpem1* more precisely. The distribution of recombinant individuals showed that SSRA5-1, SSRA7-1, INDEL-D5, INDEL-N2, and INDEL-N14 were located on the same side of *Brpem1* with SSRA1-1, but SSRA6-21, SSRA6-18, and INDEL-I8 were located on the same side as SSRA2-16 (Figure 5). In addition, both INDEL-N14 and INDEL-I8 were more tightly linked with *Brpem1* at a genetic distance of.05 cm. We mapped *Brpem1* on a 64.25-kb region, containing 13 genes between the markers INDEL-N14 and INDEL-I8.

### Identification of the Candidate Gene for *Brpem1* Based on Whole-Genome Re-Sequencing

**Figure 6:**
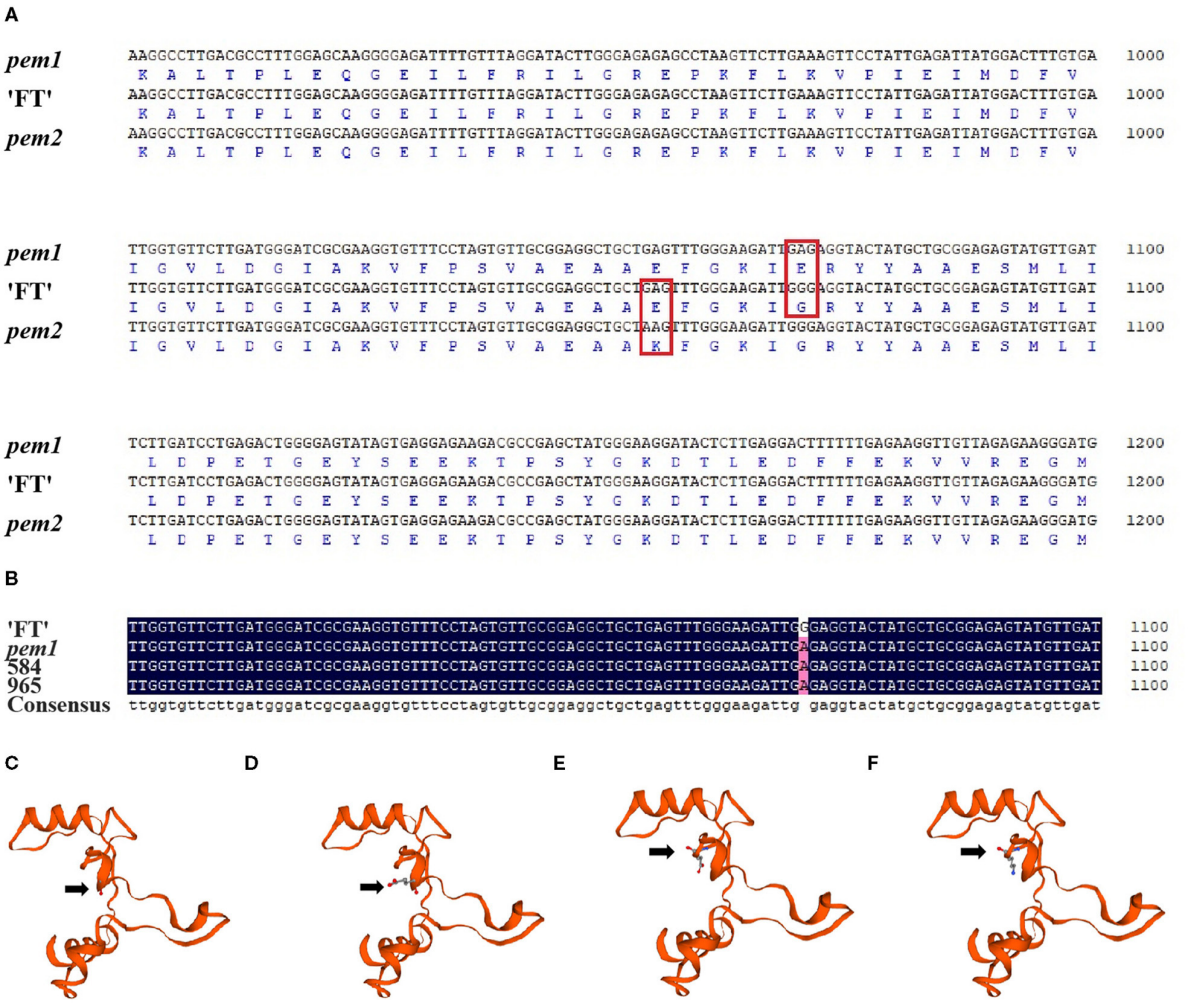
Sequence alignments and three-dimensional structure of *BraA10g021490.3C* in *pem1* and *pem2*. **(A)** Alignments of the nucleotide and amino acid sequences of *BraA10g021490.3C* in *pem1* and *pem2* with “FT.” Red box, mutation site of *pem1* and *pem2*. **(B)** Alignment of the nucleotide sequences of *BraA10g021490.3C* in two F_2_-recombined individuals compared with “FT” and *pem1*. “584” and “96” are the name of F_2_-recombined individuals. Three-dimensional structure of BraA10g021490.3C in “FT” **(C)** and *pem1* **(D)** around the mutation site of *pem1* comprises 90 amino acids. Arrows: amino acid 357 (Gly) of “FT” **(C)** and (Glu) of *pem1* **(D)**. Three-dimensional structure of BraA10g021490.3C in “FT” **(E)** and *pem2* **(F)** around the mutation site comprised 90 amino acids. Arrows: amino acid residue 352 (Glu) of “FT” **(E)** and (Lys) of *pem2* **(F)**.

Limited by mapping population size, traditional map-based cloning could not precisely locate candidate genes. The whole-genome re-sequence was conducted to detect variations between “FT” and *pem1*. After filtering out heterozygous sites, four non-synonymous SNPs were identified in the candidate region between INDEL-N14 and INDEL-I8. All the four were located on *BraA10g021490.3C* (Supplementary Table S4). Clone sequencing showed that three of the four SNPs were not stable at *pem1* and only one SNP invariably existed (Supplementary Figure S3 and Figure 6A). This SNP located on the 1,070th nucleotide of *BraA10g021490.3C* in *pem1* was GA (GGG/GAG), which caused an amino acid transformation from Gly to Glu (G to E) on amino acid residue 357 of *BraA10g021490.3C*.

According to the genetic map of *Brpem1*, two recombined individuals between the two most closely linked markers, INDEL-N14 and INDEL-I8, were used to clone sequencing. Sequencing alignment of *BraA10g021490.3C* in two recombined individuals proved that this SNP co-segregated with the pale green trait (Figure 6B). The predicted three-dimensional structure of the protein showed that the change in the amino acid in *pem1* led to a difference in the three-dimensional protein structures in the mutant site between *pem1* and “FT” (Figures 6C,D). Hence, we speculated that *BraA10g021490.3C* was the candidate gene for *Brpem1*.

### Sequence Analysis of *Brpem2*

Figure 6A and Supplementary Figure S3 show the sequencing results of *BraA10g021490.3C* in *pem2*. A single nucleotide variation (SNV) from GA (GAG/AAG) was found in nucleotide 1054 of *BraA10g021490.3C* in *pem2*, which led to a transformation from Glu to Lys (E to K) in amino acid residue 352 (Figure 6A). The three-dimensional structure of the protein is shown in Figures 6E,F. The amino acid transformation caused by the SNV also led to differences in the three-dimensional structure of the protein at the mutation site. These results suggest that *BraA10g021490.3C* was probably responsible for the pale green trait of *pem2*.

### Bioinformatic Analysis of Candidate Genes

*BraA10g021490.3C* encodes 3,8-divinyl protochlorophyllide a 8-vinyl reductase, which is a key enzyme in chlorophyll biosynthesis. Therefore, we designated *BraA10g021490.3C* as *BrDVR*. The single-copy *BrDVR* in the Chinese cabbage genome has only one 1,233-bp exon, and it encodes a mature protein of 410 amino acids. TargetP and ChloroP predicted that BrDVR has a section chloroplast transfer peptide of 58 amino acids (Supplementary Tables S7, S8). Online NCBI analysis revealed a conserved domain of BrDVR between amino acids 34 and 410 (Supplementary Figure S4A). The amino acid transformations in *pem1* (GGG/GAG: G-to-E) and *pem2* (GAG/AAG: E-to-K) were both located in the conserved domain of BrDVR.

The amino acid sequences of 11 species were aligned to understand the relationship between BrDVR and other species homologs. The phylogenetic tree showed that BrDVR has significant similarity to *B. napus, B. oleracea, R. sativus*, and *A. thaliana*, with identities of 93.04, 89.10, 86.08, and 80.51%, respectively. It was also similar to *S. lycopersicum* (66.82%), *Capsicum annuum* (66.36%), *Citrus sinensis* (68.31%), *Populus trichocarpa* (67.67%), *Oryza sativa* (55.68%), and *Zea mays* (56.84%) (Supplementary Figures S4B, S5). These results indicated that BrDVR is relatively conservative in 11 species rang from dicotyledons to monocotyledons, including fruit trees, vegetables, and crops and more closely related to the DVR of *Brassica* than to other species.

### Expression of *BrDVR*

**Figure 7:**
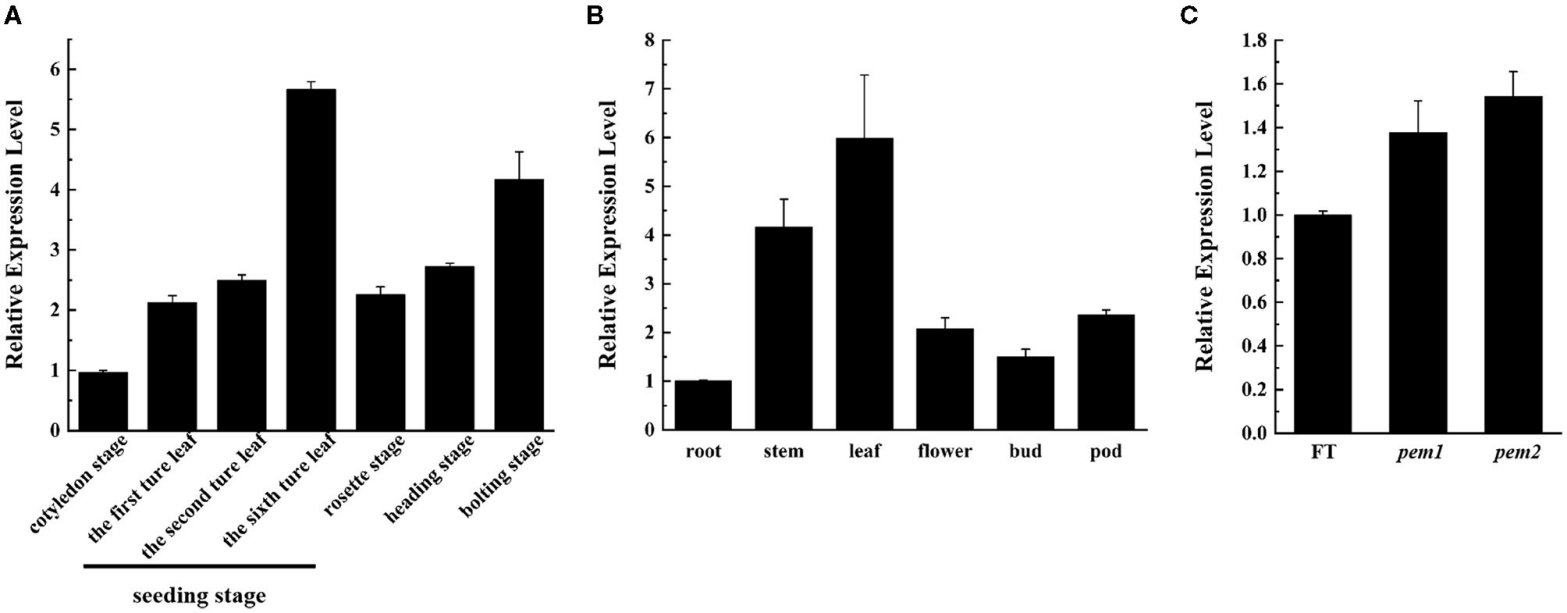
The expression level of *BrDVR*. **(A)** Expression of *BrDVR* in various organs of “FT”: root, stem, leaf, flower, bud, and pods. **(B)** Expression of *BrDVR* at different stage in “FT” leaves: cotyledon, seedling: the first, third, and sixth true leaves, rosette, heading, and bolting stages. **(C)** Expression of *BrDVR* in leaves among wild-type “FT,” *pem1* and *pem2*.

We applied qRT-PCR to investigate the spatiotemporal expression of *BrDVR* in total RNA extracted from the various organs and leaves at different stages of “FT.” The results showed that *BrDVR* was expressed mostly in the leaves, followed by the stems, pods, flowers, buds, and roots (Figure 7A). The amount of *BrDVR* expressed in leaves differed among developmental stages, being maximal during the sixth true leaf stage and minimal during cotyledon stages (Figure 7B). The *BrDVR* expression level was increased in *pem1* and *pem2* plants but was not significantly different from that in “FT” plants (Figure 7C).

### Enzyme Activity Assay

**Figure 8:**
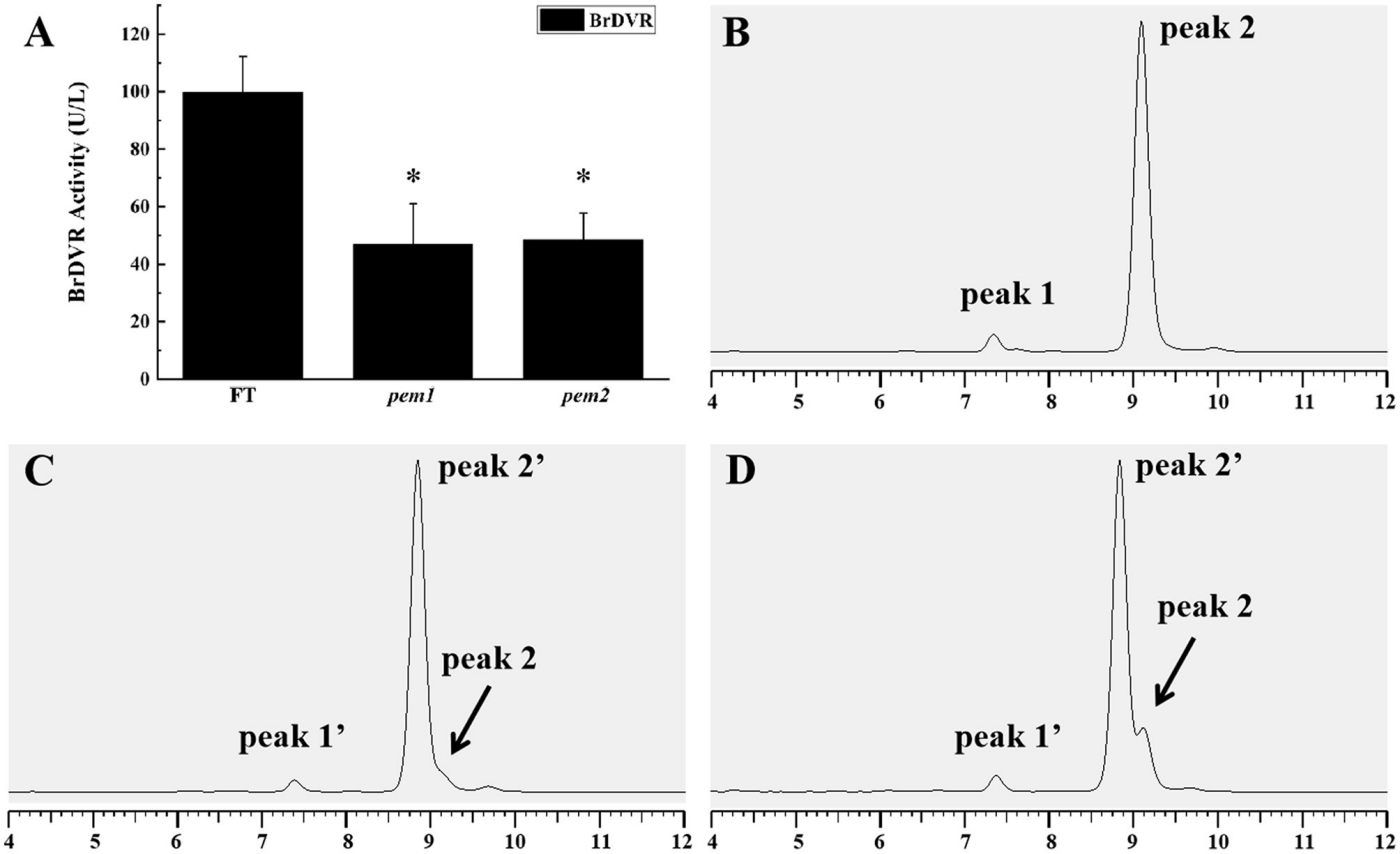
DVR activity assay and chlorophyll composition analysis among the wild-type “FT,” *pem1* and *pem2*. **(A)** DVR activity of “FT” and *pem1* and *pem2*. The asterisk represents significance. Chlorophyll composition analysis by HPLC: wild-type “FT” **(B)**, *pem1* **(C)**, *pem2* **(D)**. Peak 1, chlorophyll b; peak 2, chlorophyll a, peak 1′, chlorophyll b-like; peak 2′, chlorophyll a-like. The abscissa axis is the elution time.

DVR activity was measured via enzyme-linked immunosorbent assay (ELISA) to establish whether the SNV in *BrDVR* affects protein function. The activity of DVR did not significantly differ between *pem1* and *pem2*, but the activity in both mutants was significantly reduced to 46.94 and 48.60%, respectively, that in the wild-type “FT” (Figure 8A).

### Chlorophyll Composition Analysis

HPLC analysis found that the chlorophyll (Chl) of *pem1* and *pem2* differed from those of wild-type “FT” (Figures 8B–D). The peaks corresponding to Chl *a* and Chl *b* of the mutants were eluted a little earlier than those of the wild type. Three peaks were observed in both *pem1* and *pem2*, and peak 2' was eluted earlier than peak 2 (chlorophyll a) by approximately 15 s. The characteristics of these peaks are consistent with those of divinyl-chlorophyll (Nagata et al., 2005; Nakanishi et al., 2005; Wang et al., 2010b). The finding indicated that mutants accumulated DV-Chls and a small amount of MV-Chls.

## Discussion

Chinese cabbage takes leaves as photosynthetic and product organs, whose leaf color is an important agronomic character of Chinese cabbage. Here, we identified a pair of allele pale green mutants (Figure 3), *pem1* and *pem2*, in a 286 yellow Chinese cabbage mutant library. Based on the BSR-Seq and map-based cloning result, whole-genome re-sequencing indicated that *BrDVR* (*BraA10g021490.3C*), a homolog of *Arabidopsis* DVR, is a candidate gene for *Brpem1*. Co-segregation analysis and allele mutation analysis results verified that a single (G to A) nucleotide substitution at 1070 of *pem1* and 1054 of *pem2* caused an amino acid transformation in the conserved domain of the BrDVR protein. This is the first DVR gene identified in *Brassica* plants. Genetic, phenotypic, physiological characteristics and sequence of *pem1* and *pem2* will help to understand the biosynthesis of chlorophyll and the molecular mechanism underlying the plant pale green trait in *Brassica* plants.

Chlorophyll biosynthesis in eukaryotic organisms consists of 16 enzyme-catalyzed reactions, with DVR being the most recently discovered enzyme in chlorophyll synthesis. DVR catalyzes the reduction of the 8-vinyl group to an ethyl group on the tetrapyrrole ring and is encoded by a single copy gene in model plants, *A. thaliana* and *Oryza sativa*. Nagata et al. (2005) first identified *DVR* (AT5G18660) as indispensable for the synthesis of MV-Chls as mutation in *DVR* resulted in an accumulation of DV-Chls. At the same time, mutant DVR caused a pale green phenotype with a lower Chls content and higher Chl *a/b* ratios. Nakanishi et al. (2005) discovered that single nucleotide substitutions in *DVR* led to chloroplasts lacking obvious grana stacks and accumulated DV-Chls with only a small account of MV-Chls. Nine nucleotide deletions in *OsDVR* resulted in the mutant presenting a yellow-green phenotype, with reduced total Chl levels, sole accumulation of DV-Chls, arrested chloroplast development, and decreased output (Wang et al., 2010a). The expressive level of *BrDVR* in mutants was not significantly different from that of the wild-type “FT,” and the promoter sequences showed no variation; however, DVR activity was decreased by approximately 50%. The marked decline in DVR activity inhibits chlorophyll synthesis and lowers chlorophyll content. Similarly, mutants of *BrDVR* lead to a partial loss of protein function, with an accumulation of mainly DV-Chls and a small amount of MV-Chls, with high Chl *a*/*b* ratios (Nakanishi et al., 2005). The higher Chl *a/b* ratios caused by the absence of functional BrDVR are attributed to the unique chemical structure (Nakanishi et al., 2005). The DVR catalyzes the conversion of the vinyl group at C8 of divinyl protochlorophyllide a 8 to the ethyl group, whereas CAO catalyzes the conversion of Chl*a* to Chl*b* by converting the methyl group at C7 to the formyl group. Hence, the vinyl group side chain at C8 might interfere with the catalytic reaction of CAO at C7.

Single nucleotide substitutions in genes involved in plant biology may or may not result in large phenotypic changes. For example, Li et al. (2020) found that a single nucleotide substitution in sedoheptulose 1,7-bisphosphatase (*SBPase*) severely affects plant growth and grain yield in rice. A single nucleotide mutation (G664A) in the *TraesCS7A01G480700.1* (*chlI* of Mg-chelatase) resulted in the mutant protein losing the ability to interact with the normal protein TaCHLI-7A (Wang et al., 2020). In *A. thaliana*, a single nucleotide substitution in *DVR* leading to one amino acid substitution in the protein did a partial loss of DVR function. However, another single nucleotide substitution in *DVR* resulted in early termination of translation in *pbc2*, which eventually led to a complete loss of protein function. In the present study, mutant *BrDVR* in *pem1* and *pem2* was caused by single nucleotide substitutions (G to A), and our results show that there is a reduction in DVR activity by approximately 50% with a partial loss of DVR function. This was observed by the reduction in chlorophyll content, accumulation of DV-Chls, and higher Chl *a*/*b* ratios in the mutants compared with those in the wild type, and these differences in the pigment contributed to the pale green phenotype. Phylogenetic tree and sequence alignment illustrated that DVR is conserved in a broad range of species. The amino acid variations at 357th of *pem1* and 352th of *pem2* were located in a highly conserved region in those species (Supplementary Figure S5). Therefore, we speculated that this region is associated with protein effective function.

The allelic mutant can be used to verify the function of the mutant gene as shown in lettuce (*Lactuca sativa*) by Huo et al. (2016); sorghum (*Sorghum bicolor*) by Jiao et al. (2017) and Chinese cabbage (*Brassica rapa* ssp. *pekinensis*) by Fu et al. (2020) and so on. Fu et al. (2020) mapped two allelic early bolting mutants to *BrSDG8* in Chinese cabbage whereas Jiang et al. (2015) used two allelic mutants, *nal1-2* and *nal1-3*, to identify that *NAL1* plays a role in leaf growth and development. Zhang et al. (2018) effectively identified a round leaf shape gene in cucumber in two *round*-*leaf* (*rl*) allelic mutants. We selected seven similar pale green mutants from 286 yellow mutants in our library to analyze their allelism. It was found that the mutant genes of *pem1* and *pem2* were allelic. Mapping and sequence analysis found that a single non-synonymous nucleotide substitution (G to A) at position 1070 of *pem1* and 1054 of *pem2* in *BrDVR*. BrDVR activity assay and chlorophyll composition analysis demonstrated that impaired BrDVR led to partial loss of function, causing a pale green phenotype. Thus, we considered that *BrDVR* was responsible for the pale green phenotype of *pem1* and *pem2*.

Our experiment revealed that *BrDVR* is the caused gene for the pale green phenotype of *pem1* and *pem2*, and partial loss of its protein function is due to the single nucleotide substitution in *BrDVR*. The two allelic pale green phenotype mutants and the present study provide a good foundation for further investigation into DVR functions.

## Data Availability Statement

The raw sequence data of the BSR-Seq has been uploaded to the Sequence Read Archive at NCBI under the accession number SRR14339734. The raw sequence data of the whole genome re-sequencing has been uploaded to the Sequence Read Archive at NCBI under the accession number SRR14340669 and SRR14340670.

## Author Contributions

HF and SH conceived and designed the study. YZhao and MZ performed the experiments. YZhao wrote, reviewed, and edited the manuscript. YZhao and YZhan analyzed the data. All authors read and approved the manuscript.

## Conflict of Interest

The authors declare that the research was conducted in the absence of any commercial or financial relationships that could be construed as a potential conflict of interest.

## Publisher's Note

All claims expressed in this article are solely those of the authors and do not necessarily represent those of their affiliated organizations, or those of the publisher, the editors and the reviewers. Any product that may be evaluated in this article, or claim that may be made by its manufacturer, is not guaranteed or endorsed by the publisher.

## Supplementary Material

Supplementary Figure S1 Polymorphism screened by the primers between the two parents.

Supplementary Figure S2 Amplification of SSRA1-1 and SSR2-16 in F2 recessive individuals.

Supplementary Figure S3 Sequence of *BrDVR* based on whole-genome re-sequencing and clone sequencing.

Supplementary Figure S4 Protein sequence analysis of BrDVR and the phylogenetic tree of BrDVR and homologs.

Supplementary Figure S5 Multiple amino acid sequence alignments of BrDVR and 11 other homologous species.

Supplementary Table S1 Localization of chromosomal loci related to the pale green trait.

Supplementary Table S2 Primer sequences of SSR markers and INDEL markers.

Supplementary Table S3 Prediction of candidate genes within the gene-mapped region on chromosome A10.

Supplementary Table S4 Detection of SNP variations in the candidate region between “FT” and *pem1*.

Supplementary Table S5 Primer sequences used for clone sequencing.

Supplementary Table S6 Primer sequences for qRT-PCR.

Supplementary Table S7 The TargetP result of BrDVR.

Supplementary Table S8 The ChloroP result of BrDVR.

## References

[B1] Abyzov A. Urban A. E. Snyder M. Gerstein M. (2011). CNVnator: an approach to discover, genotype, and characterize typical and atypical CNVs from family and population genome sequencing. Genome Res. 21, 974–984. 10.1101/gr.114876.110 21324876 PMC3106330

[B2] Adra A. N. Rebeiz C. A. (1998). Chloroplast biogenesis 81. Transient formation of divinyl chlorophyll a following a 2.5 ms light flash treatment of etiolated cucumber cotyledons. Photochem. Photobiol. 68, 852–856. 10.1111/j.1751-1097.1998.tb05295.x

[B3] Beale S. I. (1999). Enzymes of chlorophyll biosynthesis. Photosynth. Res. 60, 47–73. 10.1023/A:1006297731456

[B4] Beale S. I. (2005). Green genes gleaned. Trends Plant Sci. 10, 309–312. 10.1016/j.tplants.2005.05.005 15951223

[B5] Carol P. Stevenson D. Bisanz C. Breitenbach J. Sandmann G. Mache R.. (1999). Mutations in the Arabidopsis gene *IMMUTANS* cause a variegated phenotype by inactivating a chloroplast terminal oxidase associated with phytoene desaturation. Plant Cell 11, 57–68. 10.1105/tpc.11.1.57 9878632 PMC144096

[B6] Chisholm S. W. Frankel S. L. Goericke R. Olson R. J. Palenik B. Waterbury J. B.. (1992). *Prochlorococcus marinus* nov. gen. nov. sp.: a marine prokaryote containing divinylchlorophyll a and b. Arch. Microbiol. 157, 297–300. 10.1007/BF00245165

[B7] Fan X. Abbott T. E. Larson D. Chen K. (2014). BreakDancer: identification of genomic structural variation from paired-end read mapping. Curr. Protoc. Bioinform. 45, 1–11. 10.1002/0471250953 25152801 PMC4138716

[B8] Fromme P. Melkozernov A. Jordan P. Krauss N. (2003). Structure and function of photosystem I: interaction with its soluble electron carriers and external antenna systems. FEBS Lett. 555, 40–44. 10.1016/S0014-5793(03)01124-4 14630316

[B9] Fu W. Huang S. N. Gao Y. Zhang M. D. Qu G. Y. Wang N.. (2020). Role of *BrSDG8* on bolting in Chinese cabbage (*Brassica rapa*). Theor. Appl. Genet. 133, 2937–2948. 10.1007/s00122-020-03647-4 32656681

[B10] Fu W. Ye X. L. Ren J. Li Q. Q. Du J. T. Hou A. L.. (2019). Fine mapping of *lcm1*, a gene conferring chlorophyll-deficient golden leaf in Chinese cabbage (*Brassica rapa* ssp. *pekinensis*). Mol. Breed. 39:52. 10.1007/s11032-019-0945-z

[B11] Gao M. Hu L. Li Y. Weng Y. (2016). The chlorophyll-deficient *golden leaf* mutation in cucumber is due to a single nucleotide substitution in *CsChlI* for magnesium chelatase I subunit. Theor. Appl. Genet. 129, 1961–1973. 10.1007/s00122-016-2752-9 27435733

[B12] Hansson A. Kannangara G. C. Wettstein D. V. Hasson M. (1999). Molecular basis for semi dominance of missense mutations in the XANT HA-H (42 KDa) subunit of magnesium chelaase. Proc. Natl. Acad. Sci. U.S.A. 96, 1744–1749. 10.1073/pnas.96.4.1744 9990095 PMC15580

[B13] Holm G. (1954). Chlorophyll mutation in barley. Acta Agric. Scand. 1, 457–471.

[B14] Huo H. Henry I. M. Coppoolse E. R. Verhoef-Post M. Schut J. W. Rooij H. D.. (2016). Rapid identification of lettuce seed germination mutants by bulked segregant analysis and whole genome sequencing. Plant J. 88, 345–360. 10.1111/tpj.13267 27406937

[B15] Islam M. R. Aikawa S. Midorikawa T. Kashino Y. Satoh K. Koike H. (2008). slr1923 of Synechocystis sp. PCC6803 is essential for conversion of 3,8-divinyl(proto)chlorophyll(ide) to 3-monovinyl(proto)chlorophyll(ide). Plant Physiol. 148, 1068–1081. 10.1104/pp.108.123117 18715956 PMC2556836

[B16] Ito H. Yokono M. Tanaka R. Tanaka A. (2008). Identifification of a novel vinyl reductase gene essential for the biosynthesis of monovinyl chlorophyll in *Synechocystis* sp. PCC6803. J. Biol. Chem. 283, 9002–9011. 10.1074/jbc.M708369200 18230620

[B17] Jiang D. Fang J. Lou L. Zhao J. Yuan S. Yin L.. (2015). Characterization of a null allelic mutant of the rice NAL1 gene reveals its role in regulating cell division. PLoS One 10:e0118169. 10.1371/journal.pone.0118169 25658704 PMC4320051

[B18] Jiao Y. Burow G. Gladman N. Acosta-Martinez V. Xin Z. (2017). Efficient identification of causal mutations through sequencing of bulked F_2_ from two allelic bloomless mutants of *Sorghum bicolor*. Front. Plant Sci. 8:2267. 10.3389/fpls.2017.02267 29379518 PMC5771210

[B19] Jung K. H. Hur J. Ryu C. H. Choi Y. J. Chung Y. Y. Miyao A.. (2003). Characterization of a rice chlorophyll deficient mutant using the T-DNA gene-trap system. Plant Cell Physiol. 44, 463–472. 10.1093/pcp/pcg064 12773632

[B20] Kim J. S. Rebeiz C. A. (1996). Origin of the chlorophyll a biosynthetic heterogeneity in higher plants. J. Biochem. Mol. Biol. 29, 327–334.

[B21] Kim Y. K. Lee J. Y. Cho H. S. Lee S. S. Ha H. J. Kim S.. (2005). Inactivation of organellar glutamyl- and seryl- tRNA synthetases leads to developmental arrest of chloroplasts and mitochondria in higher plants. J. Biol. Chem. 280, 37098–37106. 10.1074/jbc.M504805200 16107332

[B22] Kolossov V. L. Bohnert H. J. Rebeiz C. A. (2006). Chloroplast biogenesis 92: *in situ* screening for divinyl chlorophyll(ide) a reductase mutants by spectroflfluorometry. Anal. Biochem. 348, 192–197. 10.1016/j.ab.2005.07.031 16337140

[B23] Kumar A. M. Dieter S. (2000). Antisense *HEMA1* RNA expression inhibits heme and chlorophyll biosynthesis in Arabidopsis. Plant Physiol. 122, 49–56. 10.1104/pp.122.1.49 10631248 PMC58843

[B24] Kumar S. Stecher G. Li M. Knyaz C. Tamura K. (2018). MEGA X: molecular evolutionary genetics analysis across computing platforms. Mol. Biol. Evol. 35, 1547–1549. 10.1093/molbev/msy096 29722887 PMC5967553

[B25] Lange B. M. Ghassemian M. (2003). Genome organization in *Arabidopsis thaliana*: a survey for genes involved in isoprenoid and chlorophyll metabolism. Plant Mol. Biol. 51, 925–948. 10.1023/A:1023005504702 12777052

[B26] Lee S. Kim J. H. Yoo E. S. Lee C. H. An G. (2005). Different regulation of chlorophyll a oxygenase genes in rice. Plant Mol. Biol. 57, 805–818. 10.1007/s11103-005-2066-9 15952067

[B27] Li C. Li N. Huang R. Chen C. P. Guo J. Yang X. R.. (2020). A single nucleotide substitution at the 3'-end of SBPase gene involved in Calvin cycle severely affects plant growth and grain yield in rice. BMC Plant Biol. 20:345. 10.1186/s12870-020-02541-x 32698774 PMC7374905

[B28] Li X. Huang S. N. Liu Z. Y. Hou L. Feng H. (2019). Mutation in *EMB1923* gene promoter is associated with chlorophyll deficiency in Chinese cabbage (*Brassica campestris* ssp. *pekinensis*). Physiol. Plant 166, 909–920. 10.1111/ppl.12979 31058333

[B29] Lonosky P. M. Zhang X. S. Honavar V. G. Dobbs D. L. Fu A. Rodermel S. R. (2004). A proteomic analysis of maize chloroplast biogenesis. Plant Physiol. 134, 560–574. 10.1104/pp.103.032003 14966246 PMC344533

[B30] Nagata N. Tanaka R. Satoh S. Tanaka A. (2005). Identifification of a vinyl reductase gene for chlorophyll synthesis in *Arabidopsis thaliana* and implications for the evolution of Prochlorococcus species. Plant Cell 17, 233–240. 10.1105/tpc.104.027276 15632054 PMC544501

[B31] Nakanishi H. Nozue H. Suzuki K. Kaneko Y. Taguchi G. Hayashida N. (2005). Characterization of the *Arabidopsis thaliana* mutant *pcb2* which accumulates divinyl chlorophylls. Plant Cell Physiol. 46, 467–473. 10.1093/pcp/pci053 15695432

[B32] Oster U. Tanaka R. Tanaka A. Rüdiger W. (2000). Cloning and functional expression of the gene encoding the key enzyme for chlorophyll b biosynthesis (CAO) from *Arabidopsis thaliana*. Plant J. 21, 305–310. 10.1046/j.1365-313x.2000.00672.x 10758481

[B33] Parham R. Rebeiz C. A. (1992). Chloroplast biogenesis: [4-vinyl] chlorophyllide a reductase is a divinyl chlorophyllide a-specific, NADPH-dependent enzyme. Biochemistry 31, 8460–8464. 10.1021/bi00151a011 1390630

[B34] Parham R. Rebeiz C. A. (1995). Chloroplast biogenesis 72: a [4-vinyl] chlorophyllide a reductase assay using divinyl chlorophyllide a as an exogenous substrate. Anal. Biochem. 231, 164–169. 10.1006/abio.1995.1516 8678296

[B35] Partensky F. Hess W. R. Vaulot D. (1999). Prochlorococcus, a marine photosynthetic prokaryote of global signifificance. Microbiol. Mol. Biol. Rev. 63, 106–127. 10.0000/PMID10066832 10066832 PMC98958

[B36] Porra R. J. (1997). Recent progress in porphyrin and chlorophyll biosynthesis. Photochem. Photobiol. 63, 492–516. 10.1111/j.1751-1097.1997.tb08596.x

[B37] Preiss S. Thornber J. P. (1995). Stability of the apoproteins of lightharvesting complex I and II during biogenesis of thylakoids in the chlorophyll b-less barley mutant chlorina f2. Plant Physiol. 107, 709–717. 10.2307/4276383 12228395 PMC157186

[B38] Rebeiz C. A. Kolossov V. L. Briskin D. Gawienowski M. (2003). “Chloroplast biogenesis: chlorophyll biosynthetic heterogeneity, multiple biosynthetic routes, and biological spin-offs,” in Handbook of Photochemistry and Photobiology, ed Nalwa H. S. (Los Angeles, CA: American Scientific Publishers), 183–248.

[B39] Rudoi A. B. Shcherbakov R. A. (1998). Analysis of the chlorophyll biosynthetic system in a chlorophyll b-less barley mutant. Photosynth. Res. 58, 71–80. 10.1023/A:1006023122582

[B40] Sun B. Jiang M. Zheng H. Jian Y. Huang W. L. Yuan Q.. (2020). Color-related chlorophyll and carotenoid concentrations of Chinese kale can be altered through CRISPR/Cas9 targeted editing of the carotenoid isomerase gene *BoaCRTISO*. Hortic. Res. 7:161. 10.1038/s41438-020-00379-w 33082968 PMC7527958

[B41] Tan C. Liu Z. Y. Huang S. N. Feng H. (2019). Mapping of the male sterile mutant gene *ftms* in *Brassica rapa* L. ssp. *pekinensis* via BSR-Seq combined with whole-genome resequencing. Theor. Appl. Genet. 132, 355–370. 10.1007/s00122-018-3223-2 30382313

[B42] Tanaka A. Ito H. Tanaka R. Tanaka N. K. Yoshida K. Okada K. (1998). Chlorophyll a oxygenase (CAO) is involved in chlorophyll b formation from chlorophyll a. Proc. Natl. Acad. Sci. U.S.A. 95, 12719–12723. 10.1073/pnas.95.21.12719 9770552 PMC22897

[B43] Tripathy B. C. Rebeiz C. A. (1988). Chloroplast biogenesis 60. Conversion of divinyl protochlorophyllide to monovinyl protochlorophyllide in green (ing) barley, a dark monovinyl/light divinyl plant species. Plant Physiol. 87, 89–94. 10.1104/pp.87.1.89 16666133 PMC1054704

[B44] Wang C. J. Zhang L. L. Li Y. Z. Ali-Buttar Z. Wang N. Xie Y. Z.. (2020). Single nucleotide mutagenesis of the *TaCHLI* gene suppressed chlorophyll and fatty acid biosynthesis in common wheat seedlings. Front. Plant Sci. 11:97. 10.3389/fpls.2020.00097 32153608 PMC7046076

[B45] Wang K. Li M. Hakonarson H. (2010a). ANNOVAR: functional annotation of genetic variants from next-generation sequencing data. Nucleic Acids Res. 38:e164. 10.1093/nar/gkq603 20601685 PMC2938201

[B46] Wang P. R. Gao J. X. Wan C. M. Zhang F. T. Xu Z. J. Huang X. Q.. (2010b). Divinyl chlorophyll(ide) a can be converted to monovinyl chlorophyll(ide) a by a divinyl reductase in rice. Plant Physiol. 153, 994–1003. 10.1104/pp.110.158477 20484022 PMC2899930

[B47] Wu H. Shi N. An X. Liu C. Fu H. Cao L.. (2018). Candidate genes for yellow leaf color in common wheat (*Triticum aestivum* L.) and major related metabolic pathways according to transcriptome profiling. Int. J. Mol. Sci. 19:1594. 10.3390/ijms19061594 29843474 PMC6032196

[B48] Zapata M. Rodríguez F. Garrido J. L. (2000). Separation of chlorophylls and carotenoids from marine phytoplankton:a new HPLC method using a reversed phase C8 column and pyridine-containing mobile phases. Mar. Ecol. Prog. Ser. 195, 29–45. 10.3354/meps195029

[B49] Zhang C. W. Chen F. F. Zhao Z. Y. Hu L. L. Liu H. Q. Cheng Z. H.. (2018). Mutations in *CsPID* encoding a Ser/Thr protein kinase are responsible for round leaf shape in cucumber (*Cucumis sativus* L.). Theor. Appl. Genet. 131, 1379–1389. 10.1007/s00122-018-3084-8 29541828

[B50] Zhao Y. Wang M. L. Zhang Y. Z. Dul F. Pan T. (2008). A chlorophyll-reduced seedling mutant in oilseed rape *Brassica napus* for untilization in F1 hybrid production. Plant Breed. 119, 131–135. 10.1046/j.1439-0523.2000.00453.x

[B51] Zhu P. He L. Li Y. Huang W. Xi F. Lin L.. (2015). Correction: OTG-snpcaller: an optimized pipeline based on TMAP and GATK for SNP calling from ion torrent data. PLoS ONE 10:e0138824. 10.1371/journal.pone.0138824 26376440 PMC4574110

