# Supplementary material for: Mapping of a Pale Green Mutant Gene and Its Functional Verification by Allelic Mutations in Chinese Cabbage (Brassica rapa L. ssp. pekinensis)
Source: Front Plant Sci. 2021 Aug 12;12:699308. doi: 10.3389/fpls.2021.699308 (PMC8387703; doi:10.3389/fpls.2021.699308)
Supplement: Supplementary Figure S1 — Polymorphism screened by the primers between the two parents. [file Data_Sheet_1.docx]

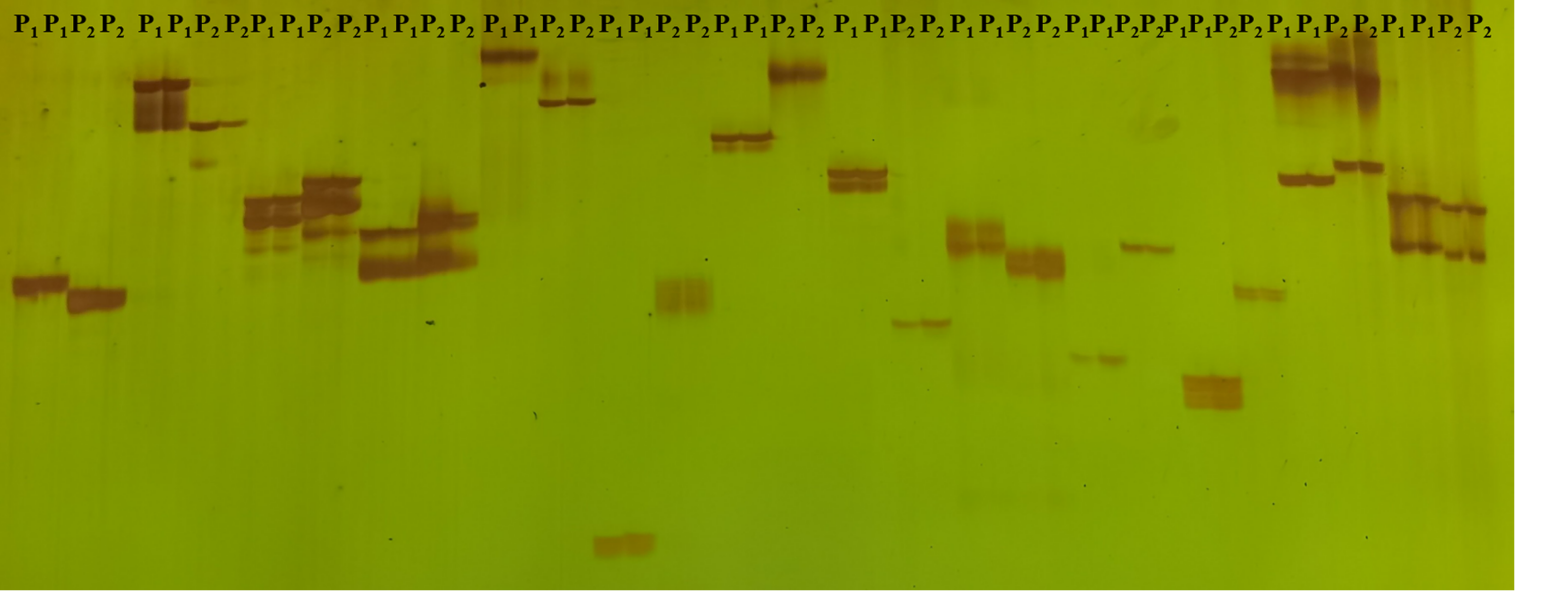


Fig. S1. Polymorphism screened by the primers between the two parents

P_1_: *pem1*, P_2_: K23


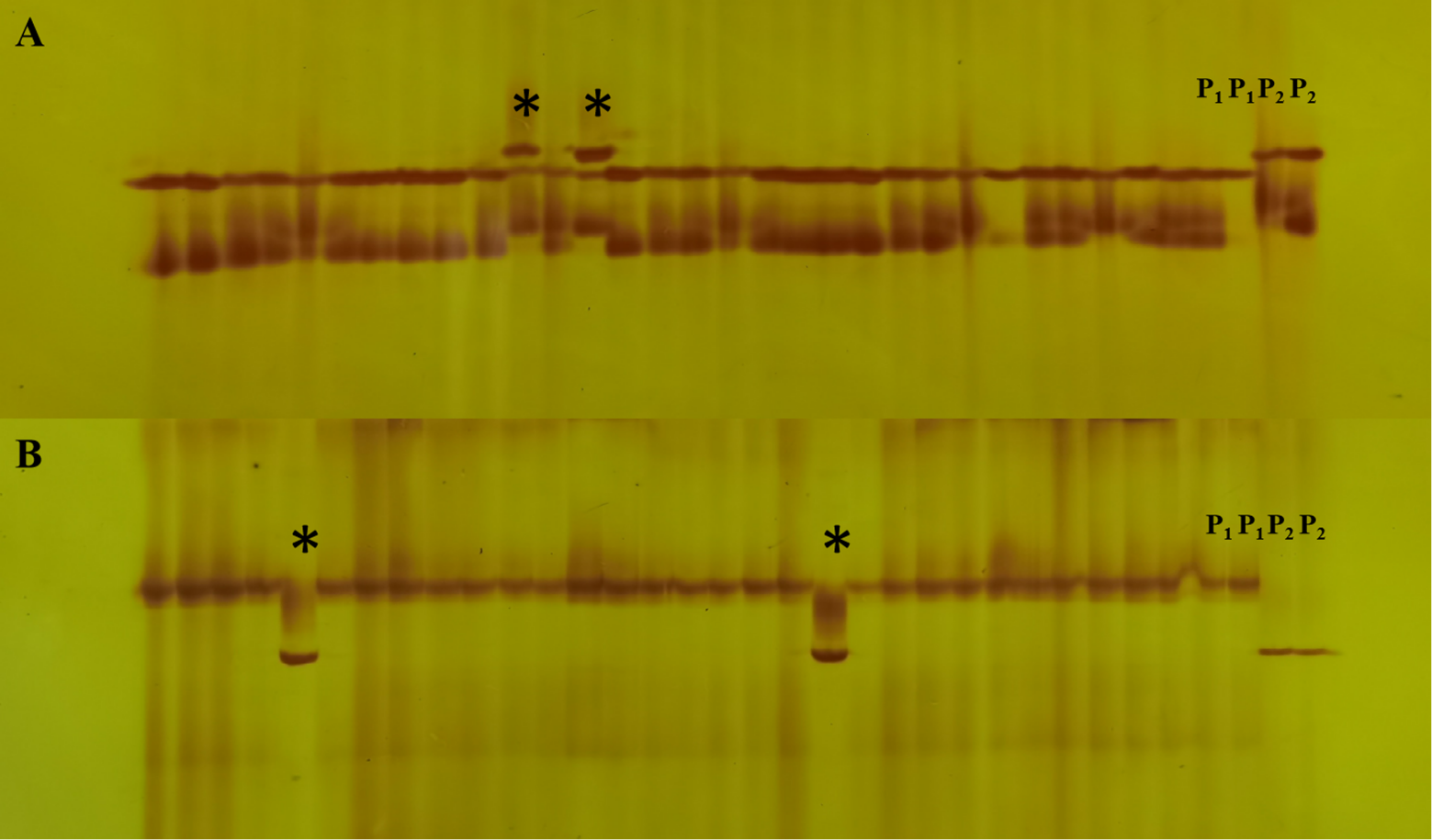


Fig. S2. Amplification of SSRA1-1 (A) and SSR2-16 (B) in F_2_ recessive individuals.

P_1_, *pem1*; P_2_, K23; *R recombinedant individuals.


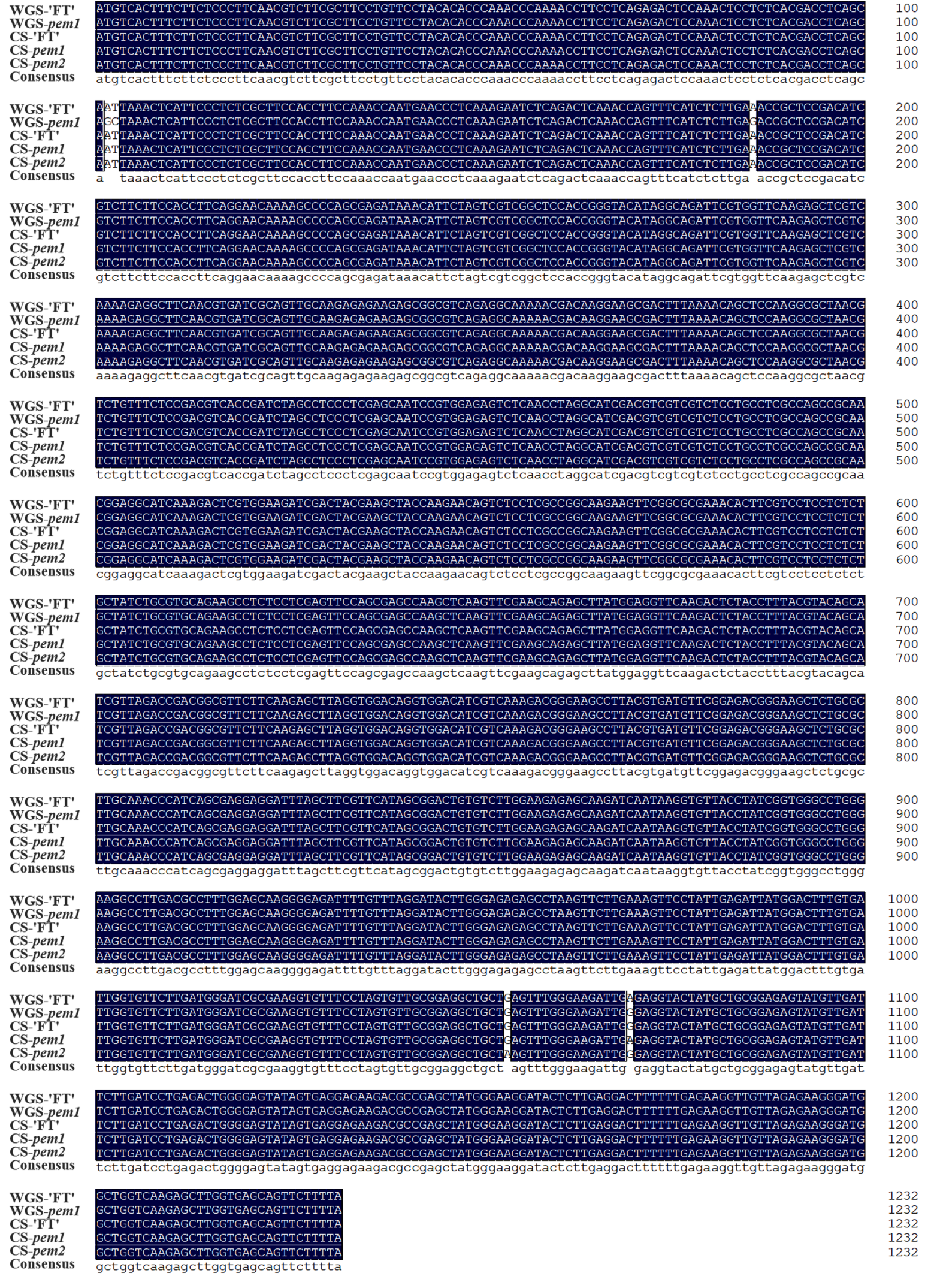


Fig. S3 Sequence of BrDVR based on whole-genome re-sequencing and clone sequencing.

Based on whole genome re-sequencing, Four non-synonymous SNPs:102^th^ G to A, 103^th^ C to T, 186^th^ G to A, 1070^th^ G to A, were detected between ‘FT’ and *pem1*. Based on clone sequencing, 1070th G to A of *pem1* and 1054th G to A of *pem2* were detected. WGS- represent sequence data from whole genome re-sequencing, CS- represent sequence data from clone sequencing.


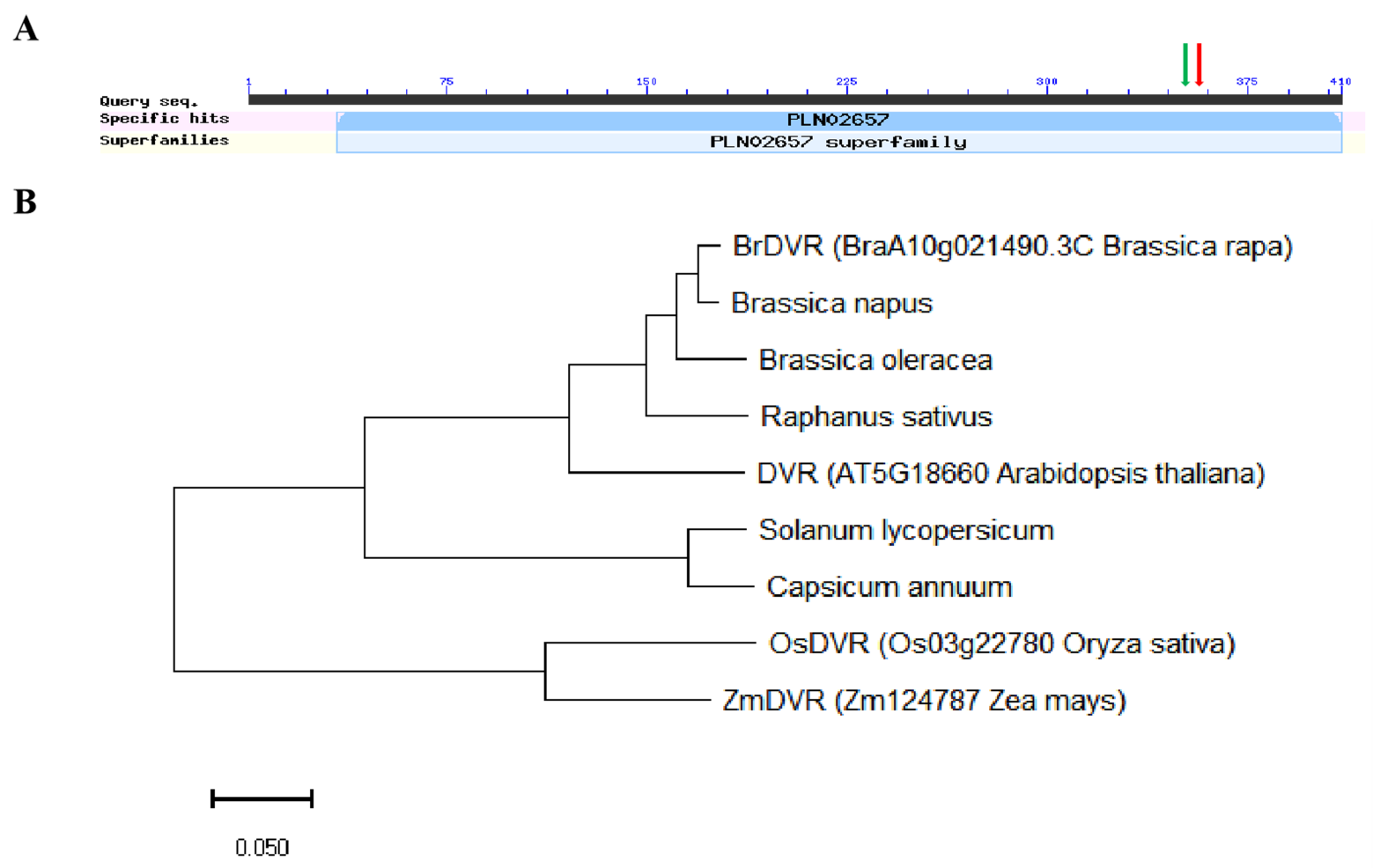


Fig. S4. Protein sequence analysis of BrDVR and phylogenetic tree of BrDVR and homologs.
(A) Conserved domain of BrDVR between amino acids 34 and 410 and mutant amino acid sites of *pem1* and *pem2* are located on the domain. PLN02657 is 3,8-divinyl protochlorophyllide a 8 -vinyl reductase. Red and green arrows, location of mutant amino acids in *pem1* and *pem2*, respectively. (B) Phylogenetic tree of BrDVR and homologs. Accession numbers of respective proteins are as follows: *B. rapa* (BrDVR, BraA10g021490.3C, XP_009120951), *B. napus* (XP_013683185), *B. oleracea* (XP_013611638), *Raphanus sativus* (XP_018443284), *A. thaliana* (DVR, At5G18660, NP_197367), *S. lycopersicum* (XP_010321216), *C. annuum* (KAF3642710), *C. sinensis* (XP_006489151), *P. trichocarpa* (XP_002311844), *O. sativa* (OsDVR, Os03g22780, ADE43128), *Z. mays* (PCB2, NP_001148282). Scale, branch length.


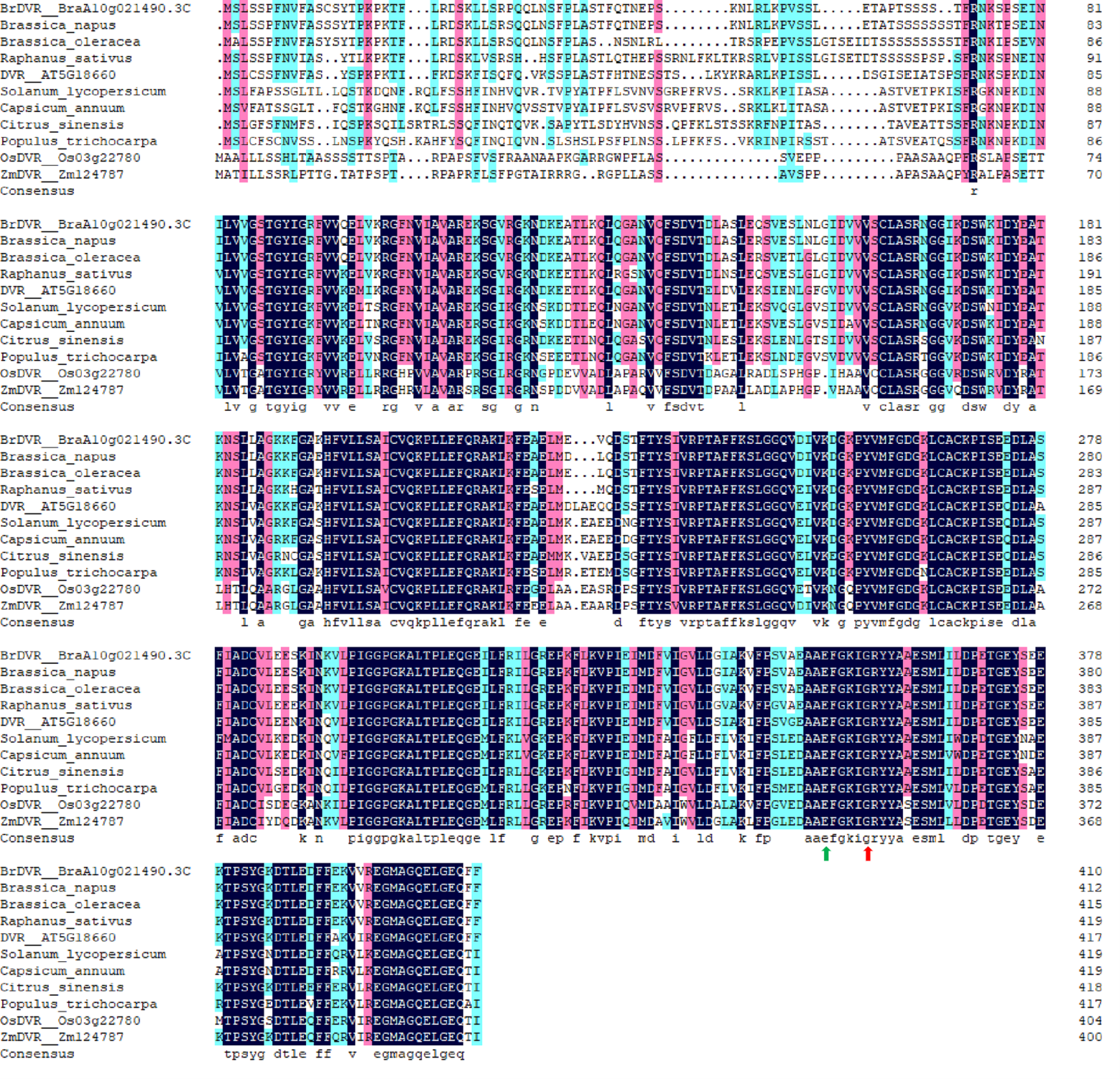


Fig. S5. Multiple amino acid sequence alignment of BrDVR and eleven other homologous species.

Aaccession numbers for the respective protein sequences are as follows: *B. rapa* (BrDVR, BraA10g021490.3C, XP_009120951), *B. napus* (XP_013683185), *B. oleracea* (XP_013611638), *R. sativus* (XP_018443284), *A. thaliana* (DVR, At5G18660, NP_197367), *S. lycopersicum* (XP_010321216), *C. annuum* (KAF3642710), *C. sinensis* (XP_006489151), *P. trichocarpa* (XP_002311844), *O. sativa* (OsDVR, Os03g22780, ADE43128), *Z. mays* (PCB2, NP_001148282). Red and green arrows, indicates the location of the mutant amino acids in *pem1* and *pem2*, respectively.
