# Supplementary material for: Mapping of a Pale Green Mutant Gene and Its Functional Verification by Allelic Mutations in Chinese Cabbage (Brassica rapa L. ssp. pekinensis)
Source: Front Plant Sci. 2021 Aug 12;12:699308. doi: 10.3389/fpls.2021.699308 (PMC8387703; doi:10.3389/fpls.2021.699308)
Supplement: Supplementary Table S1 — Localization of chromosomal loci related to the pale green trait. [file Data_Sheet_2.docx]

Table S1. Localization of chromosomal loci related to pale green trait.

| Chromosome | Start Position | End Position | Size (bp) | Total_Gene |
| --- | --- | --- | --- | --- |
| A10 | 7545866 | 9559820 | 2013954 | 307 |
| A10 | 10711277 | 15347078 | 4635801 | 905 |

Table S2. Primer sequences of SSR markers and INDEL markers.

| Marker | Forward sequence (5’-3’) | Reverse sequence (5’-3’) |
| --- | --- | --- |
| SSRA1-1 | ATACGGGTGGTGATCTCTTTG | AGTAATTTTTATTTTCTTTTTGTTTTGT |
| SSRA5-1 | ATAAAAATAGAAAATAAAAGAACGCA | CCAAACTCCTATAATTCAATACACAT |
| SSRA7-1 | CCACCGAAACAAAGAAAACA | AGGAAACGATCACGGAGATC |
| SSRA6-21 | TTTGTTCCCTGATTCTATTATTGA | TCCTGGAGTCTGATTGATCTTTA |
| SSRA2-16 | ATCTCTTGCGTAATAGATTTTGATT | GCAGTGTCTTTAGACAGGGAATT |
| SSRA6-18 | TTTTAGTTTTTTTTTTTGCTTTTGTA | TCGAAAGACGAAGCCAGAGGGAGAC |
| INDEL-D5 | ATATAAGAATGGTCACGAGCCTAA | GCAACTAGTGCAGATGAGAAGC |
| INDEL-N2 | ACATCATTCCTCACCTTCTTAGC | CACTCTCTTCCTCCACCCTCT |
| INDEL-N14 | CAAAAGCAGGAAACAACAAAGAT | TTACAGAGAAAACCAGAGAAGGC |
| INDEL-I8 | ATCCAGCTTATTTATGACTCTTCCT | ATGATATATGTCGGTGACGTTATGT |

Table S3. Prediction of candidate genes within the gene-mapped region on chromosome A10.

| Number | Gene Number | Start | End | Gene Annotations (BLASTX to Arabidopsis thaliana) | E value |
| --- | --- | --- | --- | --- | --- |
| 1 | BraA10g021420.3C | 15318924 | 15320630 | DNAJ heat shock amino-terminal domain protein | 0.0 |
| 2 | BraA10g021430.3C | 15321443 | 15324700 | DNAJ heat shock amino-terminal domain protein | 0.0 |
| 3 | BraA10g021440.3C | 15325300 | 15330835 | microtubule-associated kinase-like protein RUNKEL (RUK) | 0.0 |
| 4 | BraA10g021450.3C | 15333527 | 15333934 | arabinogalactan protein 25 | 2e-79 |
| 5 | BraA10g021460.3C | 15339347 | 15345055 | serine/threonine-protein phosphatase 6 regulatory ankyrin repeat subunit | 0.92 |
| 6 | BraA10g021470.3C | 15346296 | 15348087 | Member of TLP family of tubby like proteins that also contain an F-Box | 0.0 |
| 7 | BraA10g021480.3C | 15350838 | 15352708 | putative beta-amylase BMY3 (BMY3) | 0.0 |
| 8 | BraA10g021490.3C | 15356605 | 15357837 | 3,8-divinyl protochlorophyllide a 8-vinyl reductase | 0.0 |
| 9 | BraA10g021500.3C | 15358145 | 15360135 | RING-type E3 ubiquitin ligase | 0.0 |
| 10 | BraA10g021510.3C | 15362312 | 15364578 | alpha/beta-Hydrolases superfamily protein | 0.0 |
| 11 | BraA10g021520.3C | 15365465 | 15368022 | alpha/beta-Hydrolases superfamily protein | 0.0 |
| 12 | BraA10g021530.3C | 15368742 | 15374310 | A member of the A. thaliana imitation switch (AtISWI) subfamily of chromatin remodeling factors | 0.0 |
| 13 | BraA10g021540.3C | 15375140 | 15377394 | receptor-like cytoplasmic kinase | 0.0 |

Table S4. Detection of SNP variations in candidate region between ‘FT’ and *pem1*.

| Gene ID | Chromosome | Position | Genotype_FT | Genotype_pem1 | Location | NR |
| --- | --- | --- | --- | --- | --- | --- |
| BraA10g021490.3C | A10 | 15356706 | G | A | exon1:102 | XP_009120951.1\| PREDICTED: divinyl chlorophyllide a 8-vinyl-reductase, chloroplastic [Brassica rapa] |
| BraA10g021490.3C | A10 | 15356707 | C | T | exon1:103 | XP_009120951.1\| PREDICTED: divinyl chlorophyllide a 8-vinyl-reductase, chloroplastic [Brassica rapa] |
| BraA10g021490.3C | A10 | 15356790 | G | A | exon1:186 | XP_009120951.1\| PREDICTED: divinyl chlorophyllide a 8-vinyl-reductase, chloroplastic [Brassica rapa] |
| BraA10g021490.3C | A10 | 15357674 | G | A | exon1:1070 | XP_009120951.1\| PREDICTED: divinyl chlorophyllide a 8-vinyl-reductase, chloroplastic [Brassica rapa] |

Table S5. Primer sequences used for clone sequencing.

| Marker | Forward sequence (5’-3’) | Reverse sequence (5’-3’) |
| --- | --- | --- |
| *BrDVR*- full length | TGAGGATGAATAATAAGATCAAAGGA | TGTGAGGTAAAGCCAAGATAATGTA |
| *BrDVR*- promoter | CTTCAGGAACAAAAGCCCCA | TCGGAGAAACAGACGTTAGCG |

Table S6. Primer sequences for qRT-PCR.

| Marker | Forward sequence (5’-3’) | Reverse sequence (5’-3’) |
| --- | --- | --- |
| *ACTIN* | ATCTACGAGGGTTATGCT | CCACTGAGGACGATGTTT |
| *BrDVR*-qRT | CTTCAGGAACAAAAGCCCCA | TCGGAGAAACAGACGTTAGCG |

Table S7. TargetP result of BrDVR.

| Protein type | other | Signal peptide | Mitochondrial transfer peptide | Chloroplast transfer peptide | Thylakoid luminal transfer peptide |
| --- | --- | --- | --- | --- | --- |
| Likelihood | 0.0107 | 0 | 0.0006 | 0.9668 | 0.0219 |

Table S8. ChloroP result of BrDVR.

| Name | Length | Score | cTP  (chloroplast transit peptides) | cTP-length |
| --- | --- | --- | --- | --- |
| BrDVR | 410 | 0.589 | Y | 58 |
